# 2-Imidazole as a Substitute for the Electrophilic Group Gives Highly Potent Prolyl Oligopeptidase Inhibitors

**DOI:** 10.1021/acsmedchemlett.1c00399

**Published:** 2021-09-17

**Authors:** Henri T. Pätsi, Tommi P. Kilpeläinen, Samuli Auno, Pyry M. J. Dillemuth, Khaled Arja, Maija K. Lahtela-Kakkonen, Timo T. Myöhänen, Erik A. A. Wallén

**Affiliations:** † Drug Research Program, Division of Pharmaceutical Chemistry and Technology, Faculty of Pharmacy, University of Helsinki, P.O. Box 56, 00014 Helsinki, Finland; ‡ Drug Research Program, Division of Pharmacology and Pharmacotherapy, Faculty of Pharmacy, University of Helsinki, P.O. Box 56, 00014 Helsinki, Finland; § School of Pharmacy, Faculty of Health Sciences, University of Eastern Finland, Yliopistonranta 1C, 70211 Kuopio, Finland; ∥ Integrative Physiology and Pharmacology Unit, Institute of Biomedicine, University of Turku, Kiinanmyllynkatu 10, 20014 Turku, Finland

**Keywords:** Prolyl oligopeptidase, triazole, imidazole, pyrazole, alpha-synuclein, autophagy

## Abstract

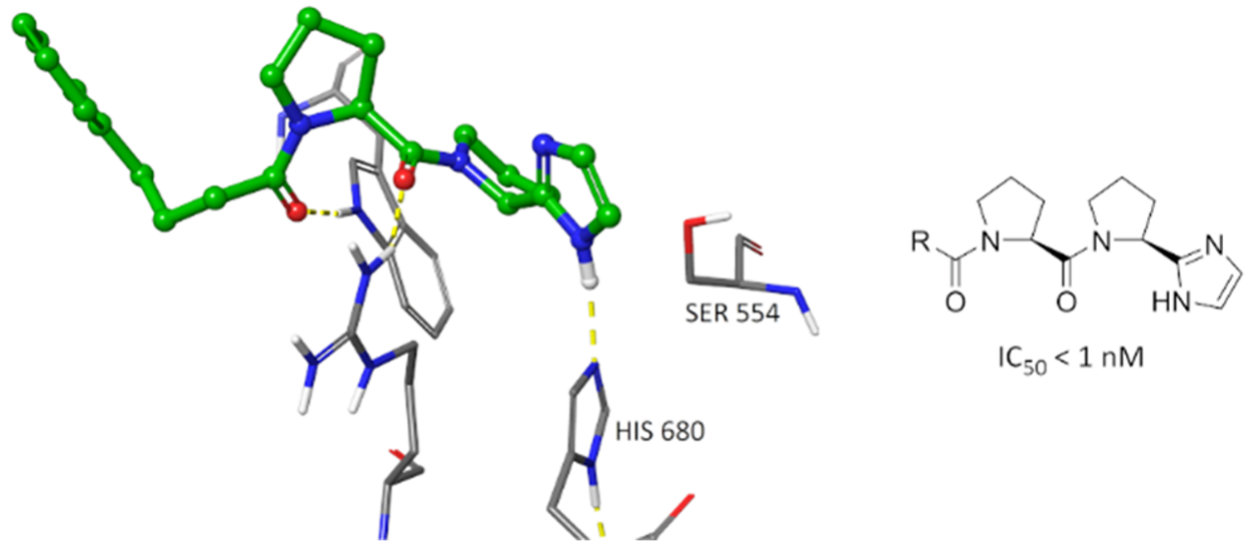

Different five-membered nitrogen-containing heteroaromatics in the position of the typical electrophilic group in prolyl oligopeptidase (PREP) inhibitors were investigated and compared to tetrazole. The 2-imidazoles were highly potent inhibitors of the proteolytic activity. The binding mode for the basic imidazole was studied by molecular docking as it was expected to differ from the acidic tetrazole. A new putative noncovalent binding mode with an interaction to His680 was found for the 2-imidazoles. Inhibition of the proteolytic activity did not correlate with the modulating effect on protein–protein-interaction-derived functions of PREP (i.e., dimerization of alpha-synuclein and autophagy). Among the highly potent PREP inhibiting 2-imidazoles, only one was also a potent modulator of PREP-catalyzed alpha-synuclein dimerization, indicating that the linker length on the opposite side of the molecule from the five-membered heteroaromatic is critical for the disconnected structure–activity relationships.

Prolyl oligopeptidase (PREP EC 3.4.21.26, also abbreviated POP) is a serine protease with endopeptidase activity, cleaving proline-containing peptides up to 30 amino acids long.^1,2^ Initially, it was suggested that PREP could regulate the degradation of proline containing neuropeptides such as substance P, arginine, vasopressin, and thyrothropin-releasing hormone (for a review, see García-Horsman et al.^3^). However, as PREP is mainly a cytosolic enzyme,^4^ its physiological role is more likely related to regulating the function of other proteins rather than cleaving neuropeptides. To support this, the effects of PREP inhibitors on neuropeptide levels have been inconclusive.^5,6^ However, inhibition of the proteolytic activity should not be overlooked as it is the mechanism of action in other therapeutic areas. Bayer has recently patented inhibitors targeting peripheral PREP for the treatment of chronic obstructive pulmonary disease^7,8^ and has a PREP inhibitor in their development pipeline.^9^

We have recently shown that PREP can regulate alpha-synuclein (αSyn) and protein phosphatase 2A (PP2A) via direct protein–protein interactions (PPIs), leading to increased αSyn aggregation^10^ and decreased autophagy,^11,12^ respectively. Small-molecular PREP inhibitors have been shown to reduce αSyn aggregation and increase the clearance of αSyn aggregates via enhanced autophagy in several *in vitro* and *in vivo* models.^11,13−15^ Thus, modulation of PREP with small-molecular ligands might be a potential therapeutic strategy to tackle several routes leading to neurodegenerative diseases related to abnormal protein processing such as Parkinson’s disease and Alzheimer’s disease. We have also demonstrated that the effects of PREP ligands on αSyn dimerization and autophagy have no direct correlation to their IC_50_ values.^15,16^ Inhibition of the proteolytic activity has been thoroughly studied, but we are still learning how protein–protein interactions of PREP are regulated. PREP is a highly dynamic protein, where inhibitor binding restricts its conformational freedom,^17^ and our working hypothesis is that the functions of PREP are dependent on what conformations PREP can adopt. The affinity of small molecules to binding sites outside the area comprised by the substrate binding pockets S1, S2, and S3 could result in different outcomes in the regulation of the conformational freedom of PREP and thereby in different regulation of its functions.

In our previous study, we showed that a tetrazole ring could be directly attached to the inhibitor structure at the position of the electrophilic group without the important carbonyl group, resulting in moderately potent inhibitors **1a**–**e**, and that the tetrazole ring was not a bioisostere of a carboxylic acid group.^16^ In addition, the tetrazoles demonstrated clearly disconnected structure–activity relationships (SARs) for inhibition of the proteolytic activity and modulation of αSyn dimerization. In the present study, other nitrogen-containing five-membered heteroaromatics with different hydrogen-bonding capabilities and charges at physiological pH were investigated. Some additional tetrazoles were also synthesized to complete the series. These compounds were also compared to a closely resembling PREP inhibitor **2a**, where both an electrophilic carbonyl group as well as a five-membered heteroaromatic are present.^18^

We demonstrate here that the 2-imidazole ring is highly superior for inhibition of the proteolytic activity of PREP compared with the other heteroaromatics. Additionally, molecular docking at the active site for the most potent inhibitors revealed a new putative noncovalent binding mode. The new compound series presented in this study also gives specific information on the disconnected SARs. Inhibition of the proteolytic activity, modulation of αSyn dimerization, and modulation of autophagy are presented for all compounds, including novel autophagy results for the previously reported tetrazoles.

The starting compounds **3**–**10** were synthesized analogously to the previously reported procedure.^16^ These were then used to synthesize the different heteroaryls according to Scheme 1. The tetrazoles **15**–**17** were synthesized from **3**–**6** according to the previously reported procedure.^16^ Compound **1a** was reacted with MeI to obtain the methylated tetrazoles **18a** and **19a**. We first made an attempt to synthesize compound **20a** by coupling 1,2,4-triazolylpyrrolidine to *N*-(4-phenylbutanoyl)-l-proline. However, as this was unsuccessful, the 1,2,4-triazoles **20a** and **20b** were instead synthesized from the corresponding prolinamides **4a** and **4b**. They were first reacted with DMF-DMA to receive an intermediate, which was then immediately reacted with N_2_H_4_.^19^ Compound **10a** was converted to compound **30a** using the same method. The alcohols **7**–**9** were oxidized to the corresponding aldehydes **21**–**23** using TEMPO and NaOCl with excellent yields.^20^ The 2-imidazoles **26**–**28** were then synthesized from the aldehydes using ammonia and glyoxal.^21^ The 4-imidazole **29a** was synthesized from the aldehyde **22a** using a TosMIC reaction.^22^ To synthesize compound **25a**, the Ohira–Bestmann reagent was first generated from dimethyl 2-oxopropylphosphonate using *p*-ABSA, which was then used to convert the aldehyde to alkyne **24a**.^23^ The alkyne was then reacted with TMSN_3_ catalyzed by CuI to acquire the 1,2,3-triazole **25a**.^24^

**Scheme 1:**
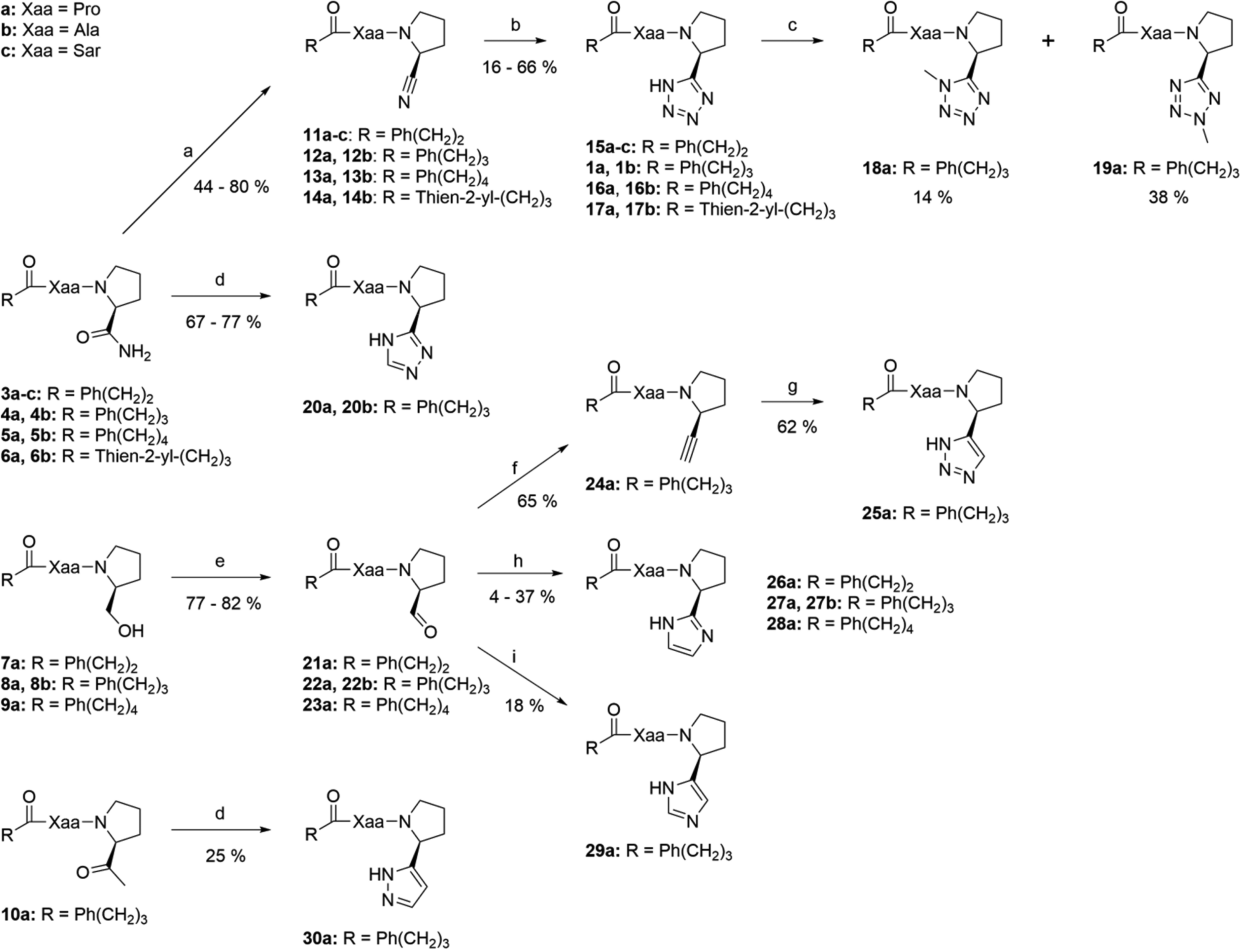
Synthesis of Heteroaryls Reagents and conditions: (a) TFAA, Et_3_N/THF, 0 °C; (b) NH_4_Cl, NaN_3_/DMF, 100 °C; (c) MeI, K_2_CO_3_/DMF; (d) (1) DMF-DMA/reflux (2) N_2_H_4_·H_2_O/AcOH, 90 °C; (e) TEMPO, NaOCl, NaBr, NaHCO_3_/DCM, 0 °C; (f) Dimethyl 2-oxopropylphosphonate, p-ABSA, K_2_CO_3_/MeCN, MeOH; (g) TMSN_3_, CuI/DMF, 100 °C; (h) NH_3_, Glyoxal/MeOH; (i) (1) TosMIC, NaCN/EtOH (2) NH_3_, reflux.

Inhibition of the proteolytic activity (IC_50_) of PREP was determined using purified recombinant porcine PREP^25^ as previously described.^16^ The results are shown in Table 1.

**Table 1:**
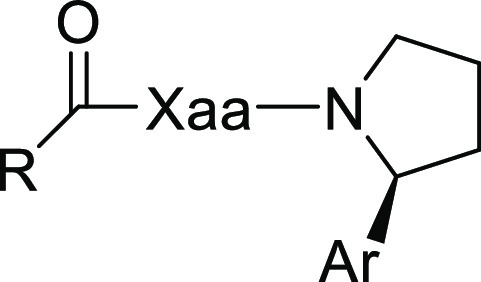
Activities of the Synthesized Compounds

compound	Xaa	Ar	R	IC_50_ (nM) (95% CI)	α-synuclein dimerization at 10 μM (%)^a^	autophagy at 10 μM (%)^b^
**KYP-2047**	l-Pro	nitrile	Ph(CH_2_)_3_-	0.2 (0.17–0.29)^c^	75^d^ (1.9)	89^d^ (3.4)
**1a**	l-Pro	tetrazolyl	Ph(CH_2_)_3_-	12 (9.9–15)^e^	82^e^ (6.8)	92 (2.3)
**1b**	l-Ala	tetrazolyl	Ph(CH_2_)_3_-	130 (71–230)^e^	75^e^ (4.9)	71 (6.2)
**1c**	Sar	tetrazolyl	Ph(CH_2_)_3_-	11 000 (8100–14 000)^e^	75^e^ (7.7)	84 (3.4)
**1d**	l-MeAla	tetrazolyl	Ph(CH_2_)_3_-	27 000 (22 000–33 000)^e^	75^e^ (7.3)	93 (4.4)
**1e**	Gly	tetrazolyl	Ph(CH_2_)_3_-	210 000^e^^,^^f^	77^e^ (6.2)	90 (6.2)
**2a**^g^	l-Pro	imidazole-2-carbonyl	Ph(CH_2_)_3_-	<1 (43%)^h^	99 (7.6)	95 (1.0)
**15a**	l-Pro	tetrazolyl	Ph(CH_2_)_2_-	1000 (790–1300)	116 (3.1)	96 (4.7)
**15b**	l-Ala	tetrazolyl	Ph(CH_2_)_2_-	3900 (3100–4800)	98 (2.4)	90 (4.7)
**15c**	Sar	tetrazolyl	Ph(CH_2_)_2_-	62 000 (35 000–110 000)	95 (10)	95 (2.7)
**16a**	l-Pro	tetrazolyl	Ph(CH_2_)_4_-	770 (540–1000)	102 (4.6)	98 (5.4)
**16b**	l-Ala	tetrazolyl	Ph(CH_2_)_4_-	5000 (4000–6100)	92 (4.7)	94 (5.7)
**17a**	l-Pro	tetrazolyl	thien-2-yl(CH_2_)_3_-	94 (57–150)	87 (9.3)	87 (5.1)
**17b**	l-Ala	tetrazolyl	thien-2-yl(CH_2_)_3_-	1100 (920–1500)	93 (5.9)	92 (5.0)
**18a**	l-Pro	1-methyltetrazolyl	Ph(CH_2_)_3_-	520 000^f^	105 (2.2)	88 (1.5)
**19a**	l-Pro	2-methyltetrazolyl	Ph(CH_2_)_3_-	12 000 (7800–17 000)	101 (5.4)	85 (2.9)
**20a**	l-Pro	1,2,4-triazolyl	Ph(CH_2_)_3_-	29 000 (21 000–40 000)	96 (4.1)	87 (4.0)
**20b**	l-Ala	1,2,4-triazolyl	Ph(CH_2_)_3_-	55 000 (30 000–100 000)	110 (3.7)	91 (5.5)
**25a**	l-Pro	1,2,3-triazolyl	Ph(CH_2_)_3_	1100 (870–1500)	104 (5.1)	93 (5.2)
**26a**	l-Pro	imidazol-2-yl	Ph(CH_2_)_2_-	<1 (41%)^h^	89 (6.3)	102 (4.3)
**27a**	l-Pro	imidazol-2-yl	Ph(CH_2_)_3_-	<1 (25%)^h^	72 (3.3)	96 (7.3)
**27b**	l-Ala	imidazol-2-yl	Ph(CH_2_)_3_-	2100 (1700–2600)	110 (2.7)	106 (5.3)
**28a**	l-Pro	imidazol-2-yl	Ph(CH_2_)_4_-	<1 (20%)^h^	110 (2.0)	102 (4.3)
**29a**	l-Pro	imidazol-4-yl	Ph(CH_2_)_3_-	44 (19–100)	98 (7.1)	92 (1.1)
**30a**	l-Pro	pyrazol-3-yl	Ph(CH_2_)_3_-	70 000 (52 000–94 000)	99 (7.1)	89 (4.0)

a Luminescence signal percentage of DMSO control with SEM.

b GFP signal percentage of DMSO control with SEM.

c Jarho et al.,^26^ measured with porcine brain homogenate.

d Kilpeläinen et al.^15^

e Kilpeläinen et al.^16^

f Confidence interval could not be determined as IC_50_ value is higher than the maximum concentration used.

g First reported by Tsutsumi et al.^18^

h PREP activity at 1 nM inhibitor concentration compared to control.

Compounds **1a** and **1b** were the two most potent tetrazoles in the previous study.^16^ Both prolonging and shortening the linker in them by one carbon atom, resulting in compounds **15a**, **15b**, **16a**, and **16b**, reduced the inhibitory activity. Replacement of the phenyl group by a 2-thienyl group, resulting in compounds **17a** and **17b**, also reduced the inhibitory activity. In all these cases the inhibitory activities were reduced by a factor in the range of 10 to 80. Furthermore, the 1- and 2-methylated tetrazoles **18a** and **19a**, respectively, had significantly lower inhibitory activities compared with the corresponding tetrazole **1a**. The two methylated tetrazoles contained about 15% of the other regioisomer (according to UPLC-MS) as they could not be fully separated from each other. As the 1-methylated tetrazole **18a** is less active, its measured activity could be mostly derived from the 2-methylated tetrazole **19a** that it contains, and the 2-methylated tetrazole **19a** may have a slightly lower IC_50_ value than indicated here.

The role of the negative charge, which is present in tetrazole-containing ligands at physiological pH, in the binding of the inhibitor was investigated by replacing the tetrazole ring of **1a** and **2a** with different triazoles. The 1,2,3-triazole **25a** was more potent than the 1,2,4-triazoles **20a** and **20b**. Nevertheless, the 1,2,3-triazole analogue of the most potent tetrazole had a lowered inhibitory activity by a factor of 100.

The basic imidazole is a highly different five-membered nitrogen-containing heteroaromatic compared with the acidic tetrazole. To our surprise, compound **27a**, which is the 2-imidazole analogue of **1a**, was more potent than the parent compound. Furthermore, modifications of the linker length, resulting in compounds **26a** and **28a**, did not have a significant effect on the inhibitory activity. The 4-imidazole analogue **29a** was slightly less potent than **1a** and the 3-pyrazolyl analogue **30a** had an IC_50_ value in the micromolar range. The purity of compound **29a** was only 86%, and the IC_50_ value should be viewed with this in mind. Interestingly, compound **27b**, the 2-imidazole analogue of **1b**, was less potent than the parent compound, indicating that the 2-imidazole ring in combination with a central alanyl residue does not result in the same favorable binding mode as with a central prolyl residue.

Docking studies were performed to gain more insight on the binding modes of the compounds containing different heteroaryls. The compounds in Table 1 were docked into the proteolytic active site of PREP.^27^ SUAM-1221,^28^ a noncovalently binding peptidic PREP inhibitor, and the cocrystallized ligand (GSK552) were docked as references. Docking was initially performed using Glide,^29^ a rigid docking method, and repeated using an induced fit docking protocol,^30^ which allows for movement in the side chains. Most of the docked compounds had a pose where the two carbonyls interact with Trp595 and Arg643 and pyrrolidine is placed in the S1 pocket (Figure 1A), as is common with most peptidic PREP inhibitors.^31^ Interestingly, the imidazole group in compound **27a** was able to act as a hydrogen bond donor to the catalytic His680 residue (Figure 1B). As in the catalytic triad, His680 further donates a hydrogen bond to Asp641. The corresponding alanine analogue, compound **27b**, did not form this interaction. Several of the compounds containing other heteroaryls could orientate similarly in the pocket, but they could not form the hydrogen bond to His680 without losing an interaction with Trp595 or Arg643. Instead, they formed hydrogen bonds to Tyr473, which is located on the opposite side of the groove (Figure 1C and Supporting Information Figures S2 and S4). Compound **27a** was also able to interact with Tyr473 instead of His680 in some poses (Supporting Information Figure S3A). The predicted binding mode of **27a** was compared to a crystal structure where KYP-2047 is covalently bound to PREP^32^ by superimposing the protein structures (Figure 1D). This showed that the five-membered heteroaryl may prevent the pyrrolidine of **27a** from fully occupying the S1 pocket, which also shifts the interaction to Arg643 compared with that of the covalently bound ligand. However, the interaction to Trp595 as well as the position of the hydrophobic chain at S3 are very similar for the two compounds.

**Figure 1:**
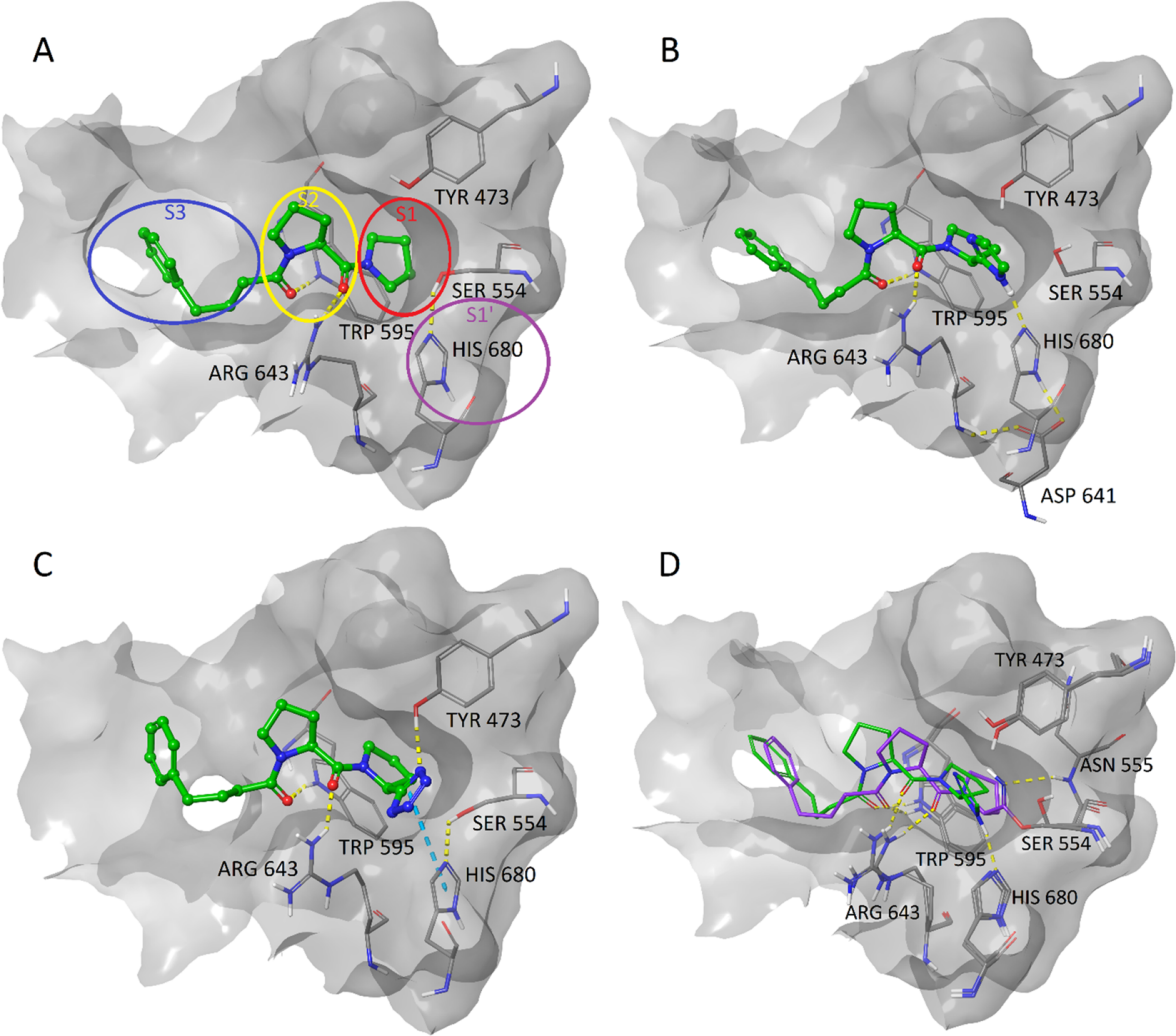
Induced fit docking poses at the proteolytic active site of PREP (PDB: 3DDU) for (A) SUAM-1221 with approximate amino acid binding sites S3–S1’ circled; (B) **27a**; (C) **1a**; (D) **27a** (green) superimposed with a crystal structure of PREP with covalently bound KYP-2047 (purple)(PDB: 4AN0). Hydrogen bonds are shown as yellow dashed lines and π–π stacking interactions as blue dashed lines.

Reference compound **2a** contains an electrophilic carbonyl group connecting the pyrrolidine and imidazole rings. The additional carbonyl group prevents the imidazole ring from orientating similarly to that of **27a**, while still maintaining its interactions to Trp595 and Arg643. However, Tsutsumi et al. hypothesized that **2a** binds covalently to Ser554 at the carbonyl, which could result in the heteroaryl positioning suitably to accept a hydrogen bond from His680.^18^ They based this theory on a previously crystallized structure, showing the interaction between a similar peptide-like compound and porcine pancreatic elastase, which is also a serine protease with a catalytic triad consisting of Ser, His, and Asp.^33^ PREP was later crystallized with covalently bound Z-prolyl-prolinal (ZPP), where a similarly positioned aldehyde and Ser554 form a hemiacetal adduct.^31^ ZPP was as potent of an inhibitor as **2a** when assayed by Tsutsumi et al.,^18^ suggesting the high inhibitory activities of these compounds are probably based on the ability to form the covalent bond. Compound **27a** and its close analogues cannot form this covalent bond and are therefore the only compounds predicted to bind to His680 in the presented manner.

The effect of the compounds on autophagy was determined by using GFP-LC3B-RFP expressing HEK-293 cells.^15,34^ The effect on αSyn dimerization was evaluated with a protein fragment complementation assay (PCA) by using N2A cells.^10,15,16^ The increase in autophagic flux was measured as a decrease in GFP signal due to increased degradation of GFP-LC3B, and the decrease in αSyn dimerization was measured as a decrease in luminescence, both compared with DMSO (Table 1). KYP-2047 was used as a reference for αSyn dimerization as it is known to significantly decrease this activity, lowering the luminescence to 75% of the DMSO control.^15^ Rapamycin and starvation are known to induce autophagy, lowering the fluorescence to 65–70% in our assays, and were therefore used as reference compounds for this. Test compounds were assayed at 10 μM concentration with 0.1% DMSO. A graphical representation of the mean values (± SEM) can be seen in the Supporting Information (Figure S1).

The tetrazoles were previously shown to decrease PREP catalyzed αSyn dimerization.^16^ The effect of these tetrazoles on autophagy was determined for the first time in this study. Compound **1b** was effective at inducing autophagy and compound **1c** had some effect, while compounds **1a**, **1d**, and **1e** had no significant activity. Varying the linker length in **1a** and **1b**, resulting in compounds **15a**, **15b**, **16a**, and **16b**, reduced the modulating activity for both αSyn dimerization and autophagy. Interestingly, substituting the phenyl group of **1a** with a 2-thienyl group, resulting in compound **17a**, maintained some activity on αSyn dimerization and slightly improved the activity in inducing autophagy. The alanine-based 2-thienyl analogue **17b** was inactive for both functions. Methylating the tetrazole in **1a** led to the two regioisomers **18a** and **19a**, which were capable of inducing autophagy but lost the activity on αSyn dimerization.

Compound **27a**, the 2-imidazole analogue of **1a**, was able to decrease αSyn dimerization more than the parent compound, reaching the same level of activity as reference compound KYP-2047. However, it had no effect on autophagy. Modifying the linker length, resulting in compounds **26a** and **28a**, led to a significant decrease in the effect on αSyn dimerization, with the shorter chain analogue **26a** still retaining some modulating activity. Compound **27b**, the 2-imidazole analogue of **1b**, did not retain either of the activities of the parent compound. Compounds **20a**, **29a**, and **30a**, the 1,2,4-triazolyl, 4-imidazole, and 3-pyrazolyl analogues of **1a**, respectively, showed some minor activity in modulating autophagy but no activity on αSyn dimerization. The lack of activity on αSyn dimerization and autophagy by reference compound **2a** was also observed.

The new results support the earlier observations for small molecular ligands, that inhibition of the proteolytic activity of PREP does not correlate with the ability to modulate other PPI mediated functions of PREP.^15^ The disconnected SARs are obvious for the 2-imidazoles **26a**, **27a**, and **28a**, where changing the linker length has a minor effect on the inhibitory activity but significantly affects the effect on αSyn dimerization. Ligand binding outside the area occupied by Suc-Gly-Pro-AMC, the substrate used in the assay for proteolytic activity, would be the most logical explanation for the disconnected SARs. It should be emphasized that modulations of PPIs are observed with a ligand concentration far below the IC_50_ value for some compounds, such as **18a**, **20a**, and **30a**, and that some highly potent inhibitors do not modulate the PPIs, such as **2a** and **28a**.

In conclusion, the 2-imidazoles demonstrate a surprisingly high inhibitory activity compared with the other heteroaromatics in the novel compound series. Binding to the active site was explored using molecular docking as the novel imidazoles lack a covalently binding electrophile and cannot bind with the same binding mode as reference compound **2a**. A possible interaction between the imidazole ring and the catalytic His680 residue was discovered, which could explain the high inhibitory activity of the 2-imidazole analogues. The imidazoles are slightly less polar, and as basic compounds they ionize in the opposite direction compared with the tetrazoles, which gives them a good starting point in drug development. The disconnected SARs between inhibition of proteolytic activity and modulation of PPIs was observed throughout the compound series, and this study identified the linker length on the opposite side of the molecule from the heteroaryl to be a highly critical structural feature for modulating PPI derived functions of PREP. This supports the hypothesis of a second binding site not interfering with the active site, where ligand binding can restrict the conformational freedom of PREP differently from ligand binding to the active site.

## Supporting Information Available

Synthesis of compounds **2**–**30**, biological experimental data, molecular docking experimental data, additional figures illustrating induced fit docking poses, and UPLC traces for the tested compounds (PDF)

## ABBREVIATIONS

AMC: 7-amino-4-methyl coumarin
αSyn: alpha-synuclein
br: broad
DMEM: Dulbecco’s Modified Eagle Medium
DMF-DMA: *N*,*N*-dimethylformamide dimethyl acetal
HDMS: hexamethyldisilazane
*p*-ABSA: 4-acetamidobenzenesulfonyl azide
PP2A: protein phosphatase 2A
PPI: protein–protein interaction
PREP: prolyl oligopeptidase
TEMPO: 2,2,6,6-tetramethylpiperidine 1-oxyl
TosMIC: *p*-toluenesulfonylmethyl isocyanide
