# Supplementary material for: 2-Imidazole as a Substitute for the Electrophilic Group Gives Highly Potent Prolyl Oligopeptidase Inhibitors
Source: ACS Med Chem Lett. 2021 Sep 17;12(10):1578–84. doi: 10.1021/acsmedchemlett.1c00399 (PMC8521653; doi:10.1021/acsmedchemlett.1c00399)
Supplement: Supplementary file 1 — ml1c00399_si_001.pdf [file ml1c00399_si_001.pdf]

Supporting Information

2-Imidazole as a substitute for the electrophilic group gives highly potent prolyl oligopeptidase inhibitors

Henri T. Pätsi,*[,1] Tommi P. Kilpeläinen,[2] Samuli Auno,[2] Pyry M. J. Dillemuth,[1] Khaled Arja,[1] Maija K. Lahtela-Kakkonen,[3] Timo T. Myöhänen,[2,3,4] and Erik A. A. Wallén[1]

[1]Drug Research Program, Division of Pharmaceutical Chemistry and Technology, Faculty of Pharmacy, University of Helsinki, P.O. Box 56, 00014 Helsinki, Finland

[2]Drug Research Program, Division of Pharmacology and Pharmacotherapy, Faculty of Pharmacy, University of Helsinki, P.O. Box 56, 00014 Helsinki, Finland

[3]School of Pharmacy, Faculty of Health Sciences, University of Eastern Finland, Yliopistonranta 1C, 70211 Kuopio, Finland

[4]Integrative Physiology and Pharmacology Unit, Institute of Biomedicine, University of Turku, Kiinanmyllynkatu 10, 20014 Turku, Finland

Table of Contents

Synthesis of compounds **2-30** S2

Measuring biological activity S22

Molecular docking method S24

Molecular docking figures S25

UPLC-MS figures S28

References S34

Synthesis of compounds **2-30**

General information

Unless otherwise specified, all reagents and solvents were obtained from commercial suppliers and used without purification. Compounds **1a** and **1b** were previously synthesized according to method G.[1] Microwave reactions were performed with fixed hold time in capped microwave vials using a Biotage Initiator+ (Biotage). Completion of reactions and purifications were monitored with TLC, which was performed on 60 F^{254} silica gel plates, using UV light (254 and 366 nm) and ninhydrin or iodine staining to detect products. Flash chromatography was performed using a Biotage Isolera One (Biotage) with silica gel 60 (40-63 µm mesh) unless otherwise specified. ^{1}H and ^{13}C NMR spectra were recorded at 400 MHz and 101 MHz, respectively, using an Ascend 400 (Bruker). $CDCl_3$ was used as the NMR solvent unless otherwise specified. Chemical shifts (δ) are reported in parts per million (ppm) with TMS or solvent residual peaks as reference. Many of the compounds contain two or more stable rotamers caused by restricted rotation along the amide bond. NMR signals for minor rotamers making up less than 10 % of the total signal are not reported, unless they affect the integration. Exact mass and purity of the tested compounds were analyzed with LC-MS, using a Waters Aquity UPLC system (Waters) and a Waters Synapt G2 HDMS mass spectrometer (Waters) via an ESI ion source in positive mode. The purity of all tested compounds was 96 % or higher, except for compound **29a**, for which it was 86 %.

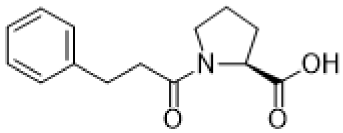

Method A: Synthesis of *N*-(3-phenylpropanoyl)-L-proline (31). 3-Phenylpropanoic acid (1.58 g, 10.5 mmol) was heated to 70 °C. $SOCl_2$ (1.2 ml, 16.5 mmol) was added dropwise. The mixture was stirred at 70 °C for 1 h followed by the evaporation of the remaining $SOCl_2$ to provide the acid chloride intermediate as an orange oil (quantitative), which was used without further purification. The acid chloride in Et_2O (7 mL) was added slowly to a solution of L-proline (1.45 g, 12.6 mmol) in a 10 % aqueous solution of Na_2CO_3 (27 mL) and Et_2O (20 mL) at 0 °C. The mixture was stirred at room temperature for 18 h before separating the phases. The aqueous phase was washed with Et_2O, acidified with 1M HCl, and extracted with Et_2O. The organic phase was washed with 0.1 M HCl, dried over anhydrous Na_2SO_4, filtered, and evaporated to provide **31** as a colourless sap (2.03 g, 78 %), which was used without further purification. The carboxylic acid used as starting material was detected as a minor impurity. ^{1}H NMR δ 11.35 (s, 1H), 7.42 – 7.14 (m, 5H), 4.71 – 4.26 (m, 1H), 3.75 – 3.27 (m, 2H), 3.01 (m, 2H), 2.83 – 2.51 (m, 2H), 2.36 – 1.86 (m, 4H). ^{13}C NMR δ 174.52, 173.58, 140.83, 128.67, 128.52, 126.45, 59.59, 47.67, 35.70, 30.99, 28.16, 24.79.

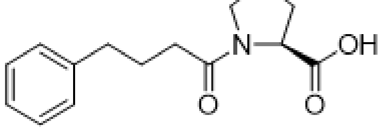

N-(4-Phenylbutanoyl)-L-proline (32). Synthesized according to method A using 4-phenylbutyric acid (5.0 g, 30.5 mmol) with a 21 h reaction time to obtain **32** as a colourless sap (7.5 g, 94 %). ^{1}H NMR δ 11.00 (s, 1H), 7.36 – 7.13 (m, 5H), 4.63 – 4.55 (m, 1H), 3.53 – 3.31 (m, 2H), 2.70 (d, *J* = 7.6 Hz, 2H), 2.36 (td, *J* = 7.3, 3.1 Hz, 3H), 2.11 – 1.93 (m, 5H). ^{13}C NMR δ 174.51, 173.67, 141.45, 128.61, 128.52, 126.13, 59.75, 47.72, 35.07, 33.46, 27.89, 25.94, 24.82.

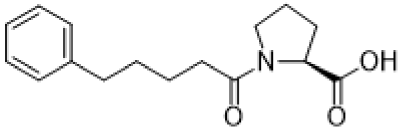

N-(5-Phenylpentanoyl)-L-proline (33). Synthesized according to method A using 5-phenylvaleric acid (2.01 g, 11.3 mmol) with an 18 h reaction time to obtain **33** as a colourless sap (2.95 g, 91 %). ^{1}H NMR δ 10.83 (s, 1H), 7.39 – 7.13 (m, 5H), 4.67 – 4.33 (m, 1H), 3.75 – 3.35 (m, 2H), 2.66 (q, 2H), 2.48 – 2.17 (m, 2H), 2.15 – 1.86 (m, 2H), 1.85 – 1.58 (m, 4H). ^{13}C NMR δ 174.69, 173.37, 142.12, 128.41, 128.34, 125.80, 59.71, 47.73, 35.71, 34.28, 30.97, 27.73, 24.75, 24.19.

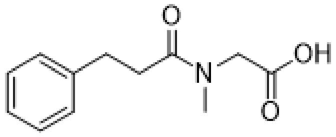

N-(3-Phenylpropanoyl)-sarcosine (34). Synthesized according to method A using 3-phenylpropanoic acid (1.01 g, 6.73 mmol) with an 22 h reaction time to obtain **34** (0.92 mg, 71 %). ^{1}H NMR δ 10.59 (s, 1H), 7.32 – 7.04 (m, 5H), 4.09 (s, 2H), 2.96 (s, 3H), 2.94 – 2.84 (m, 2H), 2.67 – 2.57 (m, 2H). ^{13}C NMR δ 174.11, 173.85, 141.12, 128.67, 128.55, 126.36, 49.84, 36.80, 35.21, 31.15.

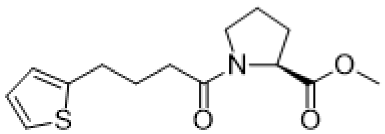

Methyl N-(4-(thien-2-yl)butanoyl)-L-prolinate (35). Synthesized according to method A using 4-(2-thienyl)butyric acid (0.73 mL, 5.0 mmol). The acid chloride intermediate with L-proline methyl ester hydrochloride (745 mg, 4.5 mmol) in DCM (15 mL) gave the crude product, which after flash chromatography (heptane/EtOAc 13:7) yielded **35** (822 mg, 65 %). ^{1}H NMR δ 7.18 – 7.08 (m, 1H), 6.99 – 6.88 (m, 1H), 6.86 – 6.81 (m, 0.85H), 6.81 – 6.78 (m, 0.15H), 4.51 (dd, *J* = 8.5, 3.8 Hz, 0.85H), 4.34 (dd, *J* = 8.6, 2.7 Hz, 0.15H), 3.75 (s, 2.55H), 3.73 (s, 0.45H), 3.68 – 3.39 (m, 2H), 2.97 – 2.85 (m, 2H), 2.47 – 1.84 (m, 8H) (two rotamers 17:3). ^{13}C NMR δ 173.07, 171.48, 144.71, 126.86, 124.63, 123.23, 58.70, 52.32, 47.05, 33.22, 29.34, 29.19, 26.49, 24.90 (additional set of signals from minor rotamer (ca. 15 %) can be seen).

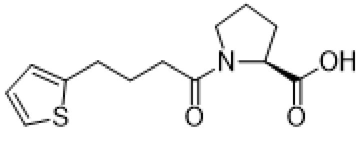

***N*-(4-(Thiophen-2-yl)butanoyl)-L-proline (36).** LiOH monohydrate (182 mg, 4.3 mmol) was added to a solution of compound **35** (813 mg, 3.0 mmol) in H_2O (8 mL) and MeOH (24 mL) and the mixture was left to stir a room temperature overnight. MeOH was evaporated and the aqueous phase was washed with DCM, acidified with 1 M HCl, and extracted with EtOAc. The organic phase was dried over anhydrous Na_2SO_4, filtered, and evaporated to provide **36** (quantitative), which was used without further purification. ^{1}H NMR δ 9.46 (s, 1H), 7.15 (dd, *J* = 5.1, 1.2 Hz, 1H), 6.94 (dd, *J* = 5.1, 3.4 Hz, 1H), 6.82 (dt, *J* = 3.4, 1.2 Hz, 1H), 4.65 – 4.54 (m, 1H), 3.59 – 3.36 (m, 2H), 2.94 (m, 2H), 2.46 – 2.35 (m, 3H), 2.14 – 1.96 (m, 5H). ^{13}C NMR δ 174.39, 173.13, 144.04, 126.85, 124.67, 123.33, 59.78, 47.72, 33.10, 28.99, 27.67, 26.23, 24.74.

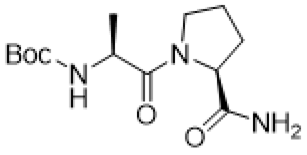

Method B: Synthesis of *N*-(*tert*-butoxycarbonyl)-L-alanyl-L-prolinamide (37). L-Prolinamide (1.0 g, 8.76 mmol) and Boc-L-Ala-OSu (2.5 g, 8.76 mmol) were dissolved in THF (35 mL) under Ar. The mixture was left to stir at room temperature for 19 h before evaporating the solvent. The residue was dissolved in EtOAc and washed with brine. Back-extractions from the aqueous phase were done with EtOAc and the combined organic phases were dried over anhydrous Na_2SO_4, filtered, and evaporated to provide the crude product, which after flash chromatography (EtOAc → EtOAc/MeOH 4:1) yielded **37** as a white foam (2.2 g, 89 %). ^{1}H NMR δ 7.43 (s, 0.1H), 6.80 (s, 0.9H), 5.71 (s, 0.1H), 5.49 (s, 0.9H), 5.34 (d, *J* = 8.2 Hz, 0.9H), 5.17 (d, *J* = 7.0 Hz, 0.1H), 4.63 (dd, *J* = 8.1, 2.7 Hz, 0.9H), 4.50 (p, *J* = 7.2 Hz, 0.9H), 4.35 – 4.30 (m, 0.1H), 4.21 – 4.16 (m, 0.1H), 3.77 – 3.50 (m, 2H), 2.58 – 2.50 (m, 0.1H), 2.40 (ddt, *J* = 12.7, 6.6, 2.8 Hz, 0.9H), 2.23 – 1.83 (m, 3H), 1.45 (s, 8.1H), 1.43 (s, 0.9H), 1.35 (d, *J* = 6.9 Hz, 2.7H), 1.32 – 1.29 (m, 0.3H) (two rotamers 9:1). ^{13}C NMR δ 173.38, 173.26, 155.32, 59.55, 47.92, 47.34, 28.49, 27.03, 25.23, 18.64 (additional signals from minor rotamer can be seen).

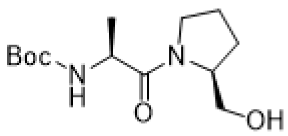

***N*-(*tert*-Butoxycarbonyl)-L-alanyl-L-prolinol (38).** Synthesized according to method B using L-Prolinol (1.5 mL, 15.4 mmol). The crude product was obtained, which after flash chromatography (hexane/EtOAc 1:4 → EtOAc/MeOH 19:1) yielded **38** (2.72 g, 65 %). ^{1}H NMR δ 5.39 (d, *J* = 8.2 Hz, 0.85H), 5.25 (d, *J* = 7.9 Hz, 0.15H), 4.76 – 4.37 (m, 2H), 4.30 – 4.16 (m, 1H), 4.02 (m, 0.3H), 3.84 – 3.29 (m, 4.7H), 2.12 – 1.79 (m, 4H), 1.60 (dq, *J* = 13.5, 6.9 Hz, 1H), 1.42 (s, 7.65H), 1.40 (s, 1.35H), 1.32 (d, *J* = 6.9 Hz, 2.25H), 1.25 (dd, *J* = 7.1, 0.9 Hz, 0.75H) (two rotamers 17:3). ^{13}C NMR δ 174.20, 155.28, 79.78, 67.02, 61.46, 48.10, 47.87, 28.47, 28.07, 24.62, 19.04 (additional set of signals from minor rotamer (ca. 15 %) can be seen).

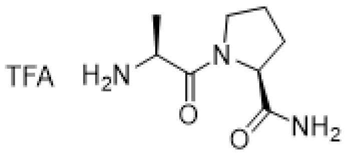

Method C: Synthesis of L-alanyl-L-prolinamide trifluoroacetate (39). TFA (4.0 mL, 52.2 mmol) was added to compound **37** (2.21 g, 7.76 mmol) in anhydrous DCM (10 mL) under Ar at 0 °C. The mixture was stirred at 0 °C for 1 h and at room temperature for 1 h before co-evaporating with methanol to provide **39** as an orange sap (quantitative), which was used without further purification. ^{1}H NMR (Methanol-d_4) δ 4.54 – 4.34 (m, 1H), 4.29 – 4.14 (m, 1H), 3.74 – 3.47 (m, 2H), 2.35 – 1.89 (m, 4H), 1.59 – 1.45 (m, 3H) (additional set of signals from minor rotamer can be seen). ^{13}C NMR (Methanol-d_4) δ 176.52, 169.64, 61.34, 49.17, 48.37, 30.81, 26.04, 16.06 (additional set of signals from minor rotamer can be seen).

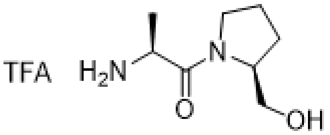

L-Alanyl-L-prolinol trifluoroacetate (40). Synthesized according to method C using **38** (2.72 g, 10.0 mmol). **40** was obtained as a pale yellow sap (quantitative). ^{1}H NMR δ 8.58 (br, 1H), 8.01 (br, 3H), 5.25 (br, 3H), 4.86 – 4.55 (m, 1H), 4.47 – 4.03 (m, 2H), 3.73 – 3.22 (m, 4H), 2.28 – 1.68 (m, 4H), 1.62 – 1.36 (m, 3H). ^{13}C NMR was not measured for crude intermediate.

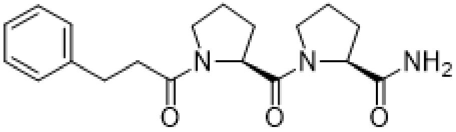

Method D: Synthesis of *N*-(3-phenylpropanoyl)-L-prolyl-L-prolinamide (3a). A solution of L-prolinamide (357 mg, 3.1 mmol) in anhydrous MeCN (18 mL) and DIPEA (1.1 mL, 6.3 mmol) was added to a solution of compound **31** (774 mg, 3.1 mmol), EDC hydrochloride (630 mg, 3.3 mmol), and HOBt hydrate (507 mg, 3.8 mmol) in anhydrous MeCN (36 mL) under argon at 0 °C. The resulting mixture was left at 0 °C without stirring for 18 h before evaporating the solvent. The residue was diluted with EtOAc, washed with a 20 % aqueous solution of citric acid, a saturated solution of $NaHCO_3$, and brine, dried over anhydrous Na_2SO_4, filtered, and evaporated to provide the crude product, which after flash chromatography (EtOAc/MeOH 9:1) yielded **3a** (854 mg, 80 %). ^{1}H NMR δ 8.23 (s, 0.5H), 7.38 – 7.17 (m, 5H), 6.93 (s, 0.5H), 5.72 (s, 0.5H), 5.41 (s, 0.5H), 4.76 – 4.57 (m, 1H), 4.49 – 4.38 (m, 0.5H), 4.36 – 4.25 (m, 0.5H), 3.93 – 3.39 (m, 4H), 3.06 – 2.89 (m, 2H), 2.74 – 2.55 (m, 2H), 2.40 – 2.30 (m, 0.5H), 2.27 – 1.73 (m, 7.5H) (two rotamers 1:1). ^{13}C NMR δ 173.95, 173.71, 172.48, 171.59, 171.09, 170.77, 141.40, 141.13, 128.66, 128.62, 128.51, 128.47, 126.35, 126.26, 60.95, 59.62, 58.84, 57.94, 47.85, 47.43, 47.40, 46.89, 36.77, 36.61, 31.67, 30.97, 30.90, 28.89, 28.69, 26.99, 25.45, 25.17, 25.00, 22.30 (double set of signals due to rotamers).

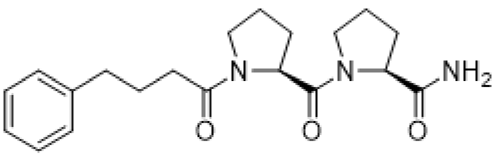

***N*-(4-Phenylbutanoyl)-L-prolyl-L-prolinamide (4a).** Synthesized according to method D using compound **32** (1.0 g, 3.83 mmol), with ethyl chloroformate (0.37 mL, 3.83 mmol) instead of EDC hydrochloride and HOBt hydrate, with a reaction time of 17 h at room temperature. The crude

product was obtained, which after flash chromatography (EtOAc/MeOH 9:1 → 1:1) yielded **4a** as a white foam (357 mg, 26 %). ^{1}H NMR δ 8.20 (s, 0.5H), 7.34 – 7.11 (m, 5H), 6.90 (s, 0.5H), 5.70 (s, 0.5H), 5.41 (s, 0.5H), 4.73 – 4.53 (m, 1H), 4.38 (dd, *J* = 7.6, 5.4 Hz, 0.5H), 4.33 – 4.23 (m, 0.5H), 3.86 – 3.36 (m, 4H), 2.74 – 2.62 (m, 2H), 2.62 – 2.53 (m, 0.5H), 2.41 – 2.23 (m, 2.5H), 2.21 – 1.71 (m, 9H) (two rotamers 1:1). ^{13}C NMR δ 173.97, 173.78, 172.56, 172.17, 171.65, 170.78, 141.81, 141.64, 128.65, 128.63, 128.45, 128.43, 126.03, 125.97, 60.92, 59.56, 58.77, 57.80, 47.81, 47.41, 47.37, 46.84, 35.28, 35.21, 33.76, 33.62, 31.63, 28.89, 28.67, 26.97, 26.17, 26.05, 25.42, 25.17, 24.99, 22.28 (double set of signals due to rotamers).

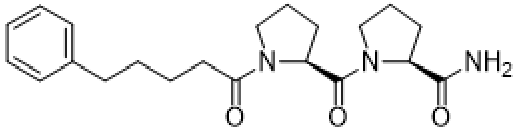

***N*-(5-Phenylpentanoyl)-L-prolyl-L-prolinamide (5a).** Synthesized according to method D using compound **33** (815 mg, 2.96 mmol). The crude product was obtained, which after flash chromatography (EtOAc → EtOAc/MeOH 4:1) yielded **5a** as a white foam (530 mg, 48 %). ^{1}H NMR δ 8.10 (s, 0.5H), 7.29 – 7.03 (m, 5H), 6.83 (s, 0.5H), 5.61 (s, 0.5H), 5.33 (s, 0.5H), 4.61 – 4.49 (m, 1H), 4.32 (dd, *J* = 7.7, 5.4 Hz, 0.5H), 4.21 (dd, *J* = 8.3, 1.4 Hz, 0.5H), 3.83 – 3.35 (m, 4H), 2.62 – 2.48 (m, 2H), 2.34 – 1.68 (m, 10H), 1.68 – 1.51 (m, 4H) (two rotamers 1:1). ^{13}C NMR δ 173.94, 173.75, 172.58, 172.34, 171.81, 170.79, 142.43, 142.33, 128.52, 128.50, 128.40, 128.37, 125.84, 125.80, 60.91, 59.55, 58.76, 57.79, 47.88, 47.42, 47.41, 46.83, 35.88, 35.78, 34.66, 34.43, 31.62, 31.27, 31.16, 28.88, 28.67, 26.96, 25.41, 25.17, 25.02, 24.41, 24.37, 22.27 (double set of signals due to rotamers).

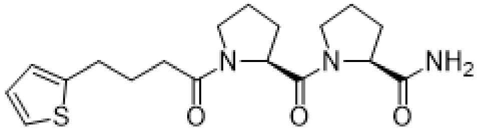

***N*-(4-(Thiophen-2-yl)butanoyl)-L-prolyl-L-prolinamide (6a).** Synthesized according to method D using compound **36** (772 mg, 2.9 mmol). The crude product was obtained, which after flash chromatography (EtOAc/MeOH 9:1) yielded **6a** (659 mg, 63 %). ^{1}H NMR δ 8.20 (s, 0.5H), 7.13 – 7.09 (m, 1H), 6.94 – 6.90 (m, 1H), 6.88 (s, 0.5H), 6.83 – 6.79 (m, 1H), 5.60 (s, 0.5H), 5.29 (s, 0.5H), 4.68 – 4.58 (m, 1H), 4.39 (dd, *J* = 7.7, 5.5 Hz, 0.5H), 4.32 – 4.24 (m, 0.5H), 3.88 – 3.43 (m, 4H), 2.93 – 2.85 (m, 2H), 2.59 (dd, *J* = 12.6, 6.3 Hz, 0.5H), 2.45 – 2.26 (m, 2.5H), 2.24 – 1.76 (m, 9H) (two rotamers 1:1). ^{13}C NMR δ 173.95, 173.64, 172.59, 171.92, 171.41, 170.76, 144.65, 144.45, 126.90, 126.88, 124.75, 124.62, 123.31, 123.25, 60.95, 59.57, 58.82, 57.85, 47.87, 47.44, 47.41, 46.87, 33.53, 33.39, 31.66, 29.34, 29.26, 28.92, 28.70, 26.89, 26.57, 26.47, 25.45, 25.19, 25.02, 22.31 (double set of signals due to rotamers).

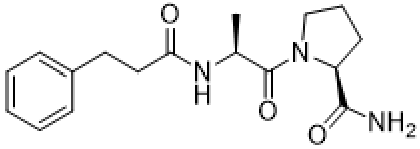

Method E: Synthesis of *N*-(3-phenylpropanoyl)-L-alanyl-L-prolinamide (3b). 3-Phenylpropionic acid (100 mg, 0.67 mmol) was heated to 70 °C. $SOCl_2$ (0.07 mL, 1.0 mmol) was added dropwise

and the mixture was stirred at 70 °C for 1 h followed by the evaporation of the remaining $SOCl_2$ to provide the acid chloride intermediate. The intermediate in DCM (1 mL) was added slowly to a solution of compound **39** (219 mg, 0.73 mmol) in Et_3N (0.46 mL, 3.3 mmol) and DCM (3 mL) at 0 °C and the mixture was stirred at room temperature for 20 h before it was washed with a 20% aqueous solution of citric acid, a saturated solution of $NaHCO_3$, and brine. Back-extractions from the aqueous phases were done with EtOAc and the combined organic phases were dried over anhydrous Na_2SO_4, filtered, and evaporated to provide the crude product, which after flash chromatography (EtOAc/MeOH 19:1 → 4:1) yielded **3b** as a white foam (115 mg, 55 %). ^{1}H NMR δ 7.32 – 7.15 (m, 5H), 6.62 (s, 0.8H), 6.42 (d, *J* = 7.6 Hz, 0.8H), 6.36 (d, *J* = 6.1 Hz, 0.2H), 6.29 (s, 0.2H), 5.88 (s, 0.2H), 5.60 (s, 0.8H), 4.75 (p, *J* = 7.0 Hz, 0.8H), 4.62 – 4.50 (m, 0.8H), 4.37 – 4.28 (m, 0.2H), 4.16 – 4.09 (m, 0.2H), 3.72 – 3.50 (m, 2H), 3.01 – 2.88 (m, 2H), 2.58 – 2.44 (m, 2H), 2.39 – 2.28 (m, 1H), 2.18 – 1.86 (m, 3H), 1.47 (d, *J* = 6.9 Hz, 0.6H), 1.29 (d, *J* = 6.9 Hz, 2.4H) (two rotamers 4:1). ^{13}C NMR δ 173.15, 172.81, 171.50, 140.69, 128.51, 128.35, 126.25, 59.52, 47.32, 46.53, 38.13, 31.54, 27.18, 25.04, 18.18 (additional set of signals from minor rotamer (ca. 20 %) can be seen).

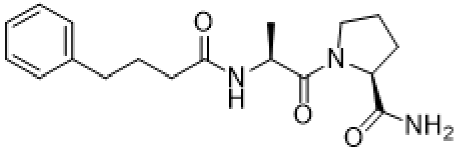

***N*-(4-Phenylbutanoyl)-L-alanyl-L-prolinamide (4b).** Synthesized according to method E using 4-phenylbutyric acid (400 mg, 2.4 mmol). The crude product was obtained, which after flash chromatography (EtOAc/MeOH 19:1 → 4:1) yielded **4b** as a white foam (433 mg, 54 %). ^{1}H NMR δ 7.61 (s, 0.2H), 7.35 – 7.12 (m, 5H), 6.68 (s, 0.8H), 6.51 (d, *J* = 7.4 Hz, 1H), 6.04 (s, 0.2H), 5.79 (s, 0.8H), 4.76 (p, *J* = 7.0 Hz, 0.8H), 4.55 (dd, *J* = 8.1, 3.1 Hz, 0.8H), 4.38 – 4.27 (m, 0.2H), 4.18 – 4.03 (m, 0.2H), 3.77 – 3.44 (m, 2H), 2.73 – 2.56 (m, 2H), 2.52 – 1.77 (m, 8H), 1.48 – 1.28 (m, 3H) (two rotamers 4:1). ^{13}C NMR δ 173.45, 172.97, 172.33, 141.57, 128.60, 128.50, 126.08, 59.68, 47.44, 46.63, 35.77, 35.32, 27.47, 27.15, 25.17, 18.27 (additional set of signals from minor rotamer can be seen).

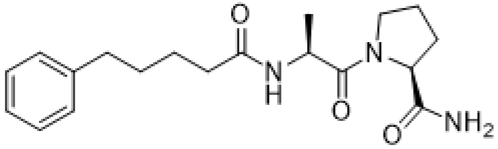

***N*-(5-Phenylpentanoyl)-L-alanyl-L-prolinamide (5b).** Synthesized according to method E using 5-phenylvaleric acid (120 mg, 0.67 mmol). The crude product was obtained, which after flash chromatography (EtOAc/MeOH 19:1 → 4:1) yielded **5b** as a white foam (152 mg, 65 %). ^{1}H NMR (Methanol-d_4) δ 7.32 – 6.99 (m, 5H), 5.53 – 5.46 (m, 0.15H), 5.48 – 5.32 (m, 0.85H), 4.61 (q, *J* = 7.0 Hz, 0.85H), 4.44 (q, *J* = 6.9 Hz, 0.15H), 3.94 – 3.74 (m, 1.7H), 3.73 – 3.46 (m, 0.3H), 2.69 – 2.50 (m, 2H), 2.47 – 1.81 (m, 6H), 1.70 – 1.51 (m, 4H), 1.33 (d, *J* = 6.9 Hz, 0.45H), 1.27 (d, *J* = 6.9 Hz, 2.55H) (two rotamers 17:3). ^{13}C NMR (Methanol-d_4) δ 175.77, 173.86, 173.50, 143.52, 129.41, 129.30, 126.74, 53.33, 48.40, 48.27, 36.55, 36.34, 32.18, 32.04, 26.42, 25.78, 16.73 (additional signals from minor rotamer (ca. 15 %) can be seen).

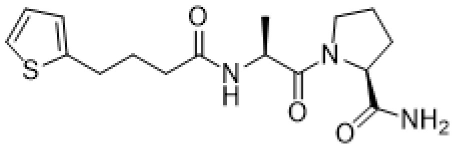

***N*-(4-(Thiophen-2-yl)butanoyl)-L-alanyl-L-prolinamide (6b).** Synthesized according to method E using 4-(2-thienyl)butyric acid (0.22 mL, 1.47 mmol). The crude product was obtained as a brown sap, which after flash chromatography (EtOAc/MeOH 19:1 → 4:1) yielded **6b** as a light brown foam (239 mg, 48 %). ^{1}H NMR δ 7.64 (s, 0.15H), 7.20 – 7.11 (m, 1H), 6.97 – 6.88 (m, 1H), 6.88 – 6.79 (m, 1H), 6.79 – 6.63 (m, 0.85H), 6.54 (s, 1H), 5.99 (s, 0.15H), 5.83 (s, 0.85H), 4.77 (p, *J* = 7.0 Hz, 0.85H), 4.64 – 4.53 (m, 0.85H), 4.42 – 4.29 (m, 0.3H), 3.79 – 3.50 (m, 2H), 2.97 – 2.79 (m, 2H), 2.57 – 2.46 (m, 0.15H), 2.39 – 2.31 (m, 0.85H), 2.31 – 2.21 (m, 2H), 2.21 – 2.09 (m, 1H), 2.08 – 1.89 (m, 4H), 1.36 (d, *J* = 6.9 Hz, 2.55H), 1.33 (d, *J* = 7.1 Hz, 0.45H) (two rotamers 17:3). ^{13}C NMR δ 173.34, 172.81, 171.93, 144.19, 126.81, 124.55, 123.22, 59.57, 47.33, 46.56, 35.33, 29.17, 27.36, 25.07, 18.18 (additional set of signals from minor rotamer (ca. 15 %) can be seen).

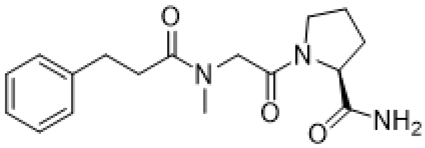

***N*-(3-Phenylpropanoyl)-sarcosinyl-L-prolinamide (3c).** Synthesized according to method D using compound **34** (900 mg, 4.07 mmol), with pivaloyl chloride (0.49 ml, 4.07 mmol) instead of EDC hydrochloride and HOBt hydrate. The crude product was obtained as a white foam, which after flash chromatography (EtOAc/MeOH 49:1 → 4:1) yielded **3c** as a white foam (603 mg, 47 %). ^{1}H NMR δ 7.28 – 7.06 (m, 5H), 6.92 (s, 0.15H), 6.80 (s, 0.85H), 5.70 (s, 0.15H), 5.34 (s, 0.85H), 4.51 (dd, *J* = 8.3, 2.4 Hz, 0.85H), 4.29 (dd, *J* = 8.5, 2.2 Hz, 0.15H), 4.12 – 4.06 (m, 1H), 3.93 – 3.85 (m, 1H), 3.69 – 3.35 (m, 2H), 3.04 (s, 3H), 2.97 – 2.80 (m, 2H), 2.69 – 2.57 (m, 2H), 2.33 – 2.22 (m, 1H), 2.17 – 1.75 (m, 3H) (two rotamers 17:3). ^{13}C NMR δ 173.59, 173.44, 168.61, 141.25, 128.62, 128.49, 126.28, 60.04, 50.84, 46.88, 37.58, 35.12, 31.07, 28.07, 24.89 (additional signals from minor rotamer (ca. 15 %) can be seen).

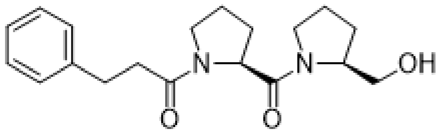

Method F: Synthesis of *N*-(3-phenylpropanoyl)-L-prolyl-L-prolinol (7a). Pivaloyl chloride (0.99 mL, 8.1 mmol) was added to a solution of compound **31** (2.00 g, 8.1 mmol) and Et_3N (1.24 mL, 8.9 mmol) in anhydrous DCM (50 mL) under argon at 0 °C. The resulting mixture was stirred for 1 h at 0 °C before the addition of L-prolinol (0.80 mL, 8.1 mmol) and Et_3N (1.24 mL, 8.9 mmol) in anhydrous DCM (10 mL). The flask was raised to room temperature and stirring was continued for another 18 h. The mixture was diluted with DCM and washed with a 20 % aqueous solution of citric acid, a saturated solution of $NaHCO_3$, and brine, dried over anhydrous Na_2SO_4, filtered, and evaporated to provide the crude product as a pale yellow sap, which after flash chromatography (EtOAc/MeOH 49:1 → 4:1) yielded **7a** as a colourless sap (1.38 g, 52 %). ^{1}H NMR δ 7.46 – 7.05 (m, 5H), 5.23 (s, 0.5H), 5.14 (dd, *J* = 8.0, 5.0 Hz, 0.5H), 4.85 (s, 0.5H), 4.68 (dd, *J* = 8.1, 4.0 Hz, 0.5H), 4.35 (tdd, *J* = 8.4, 5.7, 2.8 Hz, 0.5H), 4.23 – 4.14 (m, 0.5H), 4.00 (dt, *J* = 9.8, 6.9

Hz, 0.5H), 3.85 – 3.74 (m, 0.5H), 3.73 – 3.35 (m, 5H), 3.09 – 2.89 (m, 2H), 2.81 – 2.50 (m, 2H), 2.34 – 1.82 (m, 7H), 1.78 – 1.68 (m, 0.5H), 1.64 – 1.54 (m, 0.5H) (two rotamers 1:1). ^{13}C NMR δ 174.07, 171.85, 171.42, 171.06, 141.43, 141.35, 128.60, 128.59, 128.50, 128.49, 126.22, 126.21, 67.78, 66.96, 61.31, 60.11, 58.53, 57.90, 48.06, 48.03, 47.37, 45.84, 36.86, 36.59, 30.91, 30.88, 29.38, 29.35, 29.22, 28.02, 25.13, 24.93, 24.74, 22.39. (double set of signals due to rotamers).

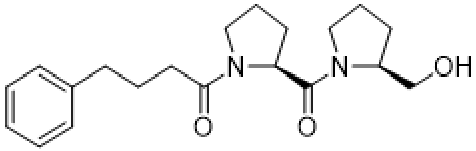

***N*-(4-Phenylbutanoyl)-L-prolyl-L-prolinol (8a).** Synthesized according to method F using compound **32** (1.97 g, 7.54 mmol) and L-prolinol (0.82 mL, 8.29 mmol) with a reaction time of 21 h. The crude product was obtained as a pale yellow oil, which after flash chromatography (EtOAc/MeOH 99:1 → 4:1) yielded **8a** as a colourless sap (1.95 g, 75 %). ^{1}H NMR δ 7.35 – 7.09 (m, 5H), 5.11 (dd, *J* = 8.0, 5.3 Hz, 0.5H), 5.83 – 4.50 (s, 1H), 4.65 (dd, *J* = 8.2, 3.9 Hz, 0.5H), 4.31 (tdd, *J* = 8.4, 5.7, 2.8 Hz, 0.5H), 4.19 – 4.11 (m, 0.5H), 3.96 (dt, *J* = 9.7, 6.9 Hz, 0.5H), 3.79 – 3.70 (m, 0.5H), 3.69 – 3.40 (m, 5H), 2.74 – 2.57 (m, 2H), 2.40 – 1.76 (m, 11H), 1.74 – 1.65 (m, 0.5H), 1.57 (dt, *J* = 12.4, 6.3 Hz, 0.5H) (two rotamers 1:1). ^{13}C NMR δ 174.10, 172.46, 171.65, 171.44, 141.82, 141.76, 128.65, 128.62, 128.39, 128.39, 125.92, 125.92, 67.72, 66.93, 61.23, 60.07, 58.45, 57.82, 48.02, 47.99, 47.33, 45.82, 35.23, 35.15, 33.72, 33.58, 29.36, 29.33, 29.20, 27.98, 26.03, 26.01, 25.12, 24.92, 24.71, 22.37 (double set of signals due to rotamers).

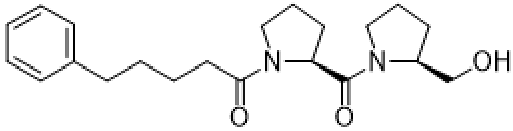

***N*-(5-Phenylpentanoyl)-L-prolyl-L-prolinol (9a).** Synthesized according to method F using compound **33** (2.93 g, 10.6 mmol) and L-prolinol (1.1 mL, 10.6 mmol) with a reaction time of 18 h. The crude product was obtained as a pale yellow sap, which after flash chromatography (EtOAc/MeOH 49:1 → 4:1) yielded **9a** as a colourless sap (2.70 g, 71 %). ^{1}H NMR δ 7.31 – 7.11 (m, 5H), 5.24 (d, *J* = 11.1 Hz, 0.5H), 5.11 (dd, *J* = 8.0, 5.4 Hz, 0.5H), 4.87 (s, 0.5H), 4.65 (dd, *J* = 8.4, 3.8 Hz, 0.5H), 4.32 (tdd, *J* = 8.4, 5.7, 2.8 Hz, 0.5H), 4.19 – 4.11 (m, 0.5H), 3.97 (dt, *J* = 9.8, 6.9 Hz, 0.5H), 3.81 – 3.71 (m, 0.5H), 3.70 – 3.38 (m, 5H), 2.72 – 2.55 (m, 2H), 2.43 – 2.06 (m, 4H), 2.04 – 1.78 (m, 5H), 1.78 – 1.53 (m, 5H) (two rotamers 1:1). ^{13}C NMR δ 174.09, 172.56, 171.76, 171.41, 142.41, 142.39, 128.48, 128.47, 128.32 (two signals), 125.73, 125.73, 67.68, 66.88, 61.19, 60.02, 58.41, 57.78, 48.06, 47.96, 47.36, 45.77, 35.85, 35.79, 34.64, 34.37, 31.21, 31.14, 29.33, 29.31, 29.18, 27.95, 25.11, 24.92, 24.69, 24.34, 24.28, 22.33 (double set of signals due to rotamers).

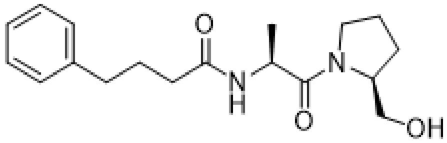

***N*-(4-Phenylbutanoyl)-L-alanyl-L-prolinol (8b).** Synthesized according to method E using 4-phenylbutyric acid (0.85 g, 5.5 mmol), with compound **40** (1.25 g, 4.4 mmol) instead of compound

39. The crude product was obtained, which after flash chromatography (hexane/acetone 3:2 → acetone) yielded **8b** (577 mg, 42 %). ^{1}H NMR δ 7.34 – 7.11 (m, 5H), 6.51 (d, J = 7.6 Hz, 0.8H), 6.45 (d, J = 7.1 Hz, 0.2H), 4.96 (p, J = 7.0 Hz, 0.2H), 4.76 (p, J = 7.0 Hz, 0.8H), 4.66 – 4.27 (m, 1H), 4.27 – 4.15 (m, 0.8H), 4.07 – 4.00 (m, 0.2H), 3.81 – 3.39 (m, 4H), 2.72 – 2.56 (m, 2H), 2.24 – 2.17 (m, 2H), 2.13 – 1.80 (m, 5.2H), 1.70 – 1.57 (m, 0.8H), 1.35 (d, J = 6.8 Hz, 2.4H), 1.29 (d, J = 6.9 Hz, 0.6H) (two rotamers 4:1). ^{13}C NMR δ 173.71, 172.07, 141.46, 128.50, 128.39, 125.96, 66.67, 61.33, 47.83, 46.74, 35.76, 35.21, 27.91, 27.07, 24.49, 18.68 (additional set of signals from minor rotamer (ca. 20 %) can be seen).

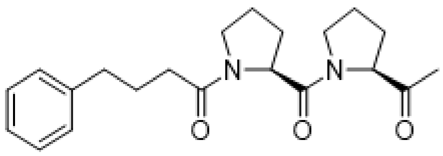

***N*-(4-Phenylbutanoyl)-L-prolyl-2(S)-acetylpyrrolidine (10a).** TFA (1.0 mL, 13 mmol) was added to a solution of tert-butyl (S)-2-acetylpyrrolidine-1-carboxylate (210 mg, 0.98 mmol) in anhydrous DCM (2.5 mL) under argon at 0 °C. The mixture was stirred for 30 minutes at 0 °C and 30 minutes at room temperature. The resulting solution was co-evaporated with MeOH to afford an orange oil, which was used without further purification. Compound **32** (309 mg, 1.18 mmol), HATU (449 mg, 1.18 mmol), and DIPEA (0.51 mL, 2.95 mmol) were added consecutively to a solution of the intermediate in anhydrous DMF (6 mL) under argon. The mixture was stirred for 2 h at room temperature before diluting with EtOAc. The organic phase was washed with a 20% aqueous solution of citric acid, a saturated solution of $NaHCO_3$, and brine. Back-extractions from the aqueous phases were done with EtOAc and the combined organic phases were dried over anhydrous Na_2SO_4, filtered, and evaporated to provide the crude product as an orange oil, which after flash chromatography (EtOAc/MeOH 99:1 → 9:1) yielded **10a** as a colourless sap (122 mg, 35 %). ^{1}H NMR δ 7.31 – 7.11 (m, 5H), 4.71 – 4.61 (m, 2H), 3.94 – 3.84 (m, 1H), 3.63 – 3.54 (m, 2H), 3.46 – 3.36 (m, 1H), 2.72 – 2.61 (m, 2H), 2.38 – 2.22 (m, 2H), 2.19 (s, 3H), 2.18 – 1.75 (m, 10H). ^{13}C NMR δ 206.87, 171.59, 170.91, 141.91, 128.64, 128.40, 125.91, 64.82, 57.56, 47.34, 47.03, 35.32, 33.65, 28.64, 27.72, 27.61, 26.03, 25.08, 24.82.

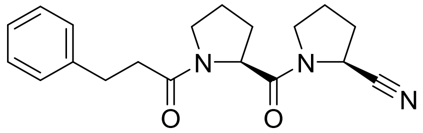

Method G: Synthesis of *N*-(3-phenylpropanoyl)-L-prolyl-2(S)-cyanopyrrolidine (11a). TFAA (0.59 mL, 4.2 mmol) was added to a solution of compound **3a** (1.22 g, 3.6 mmol) in anhydrous THF (120 mL) and Et_3N (1.2 mL, 8.5 mmol) under Ar at 0 °C. The mixture was left to stir at 0 °C for 2 h before quenching with H_2O. The solution was diluted with EtOAc, washed with a 10 % aqueous solution of citric acid, a saturated solution of $NaHCO_3$, and brine, dried over anhydrous Na_2SO_4, filtered, and evaporated to provide the crude product, which after flash chromatography (EtOAc/MeOH 9:1) yielded **11a** (748 mg, 65 %). ^{1}H NMR δ 7.37 – 7.03 (m, 5H), 4.92 – 4.79 (m, 1H), 4.57 (dd, *J* = 8.3, 4.1 Hz, 1H), 3.99 – 3.84 (m, 1H), 3.71 – 3.57 (m, 2H), 3.50 – 3.34 (m, 1H), 3.03 – 2.86 (m, 2H), 2.75 – 2.45 (m, 2H), 2.33 – 1.87 (m, 8H). ^{13}C NMR δ 171.46, 171.19, 141.33,

128.64, 128.48, 126.28, 118.75, 57.54, 47.39, 46.64, 46.50, 36.57, 30.90, 29.84, 28.87, 25.51, 25.02.

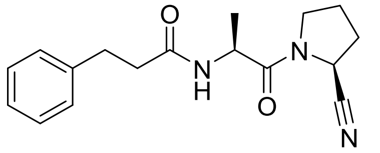

N-(3-Phenylpropanoyl)-L-alanyl-2(S)-cyanopyrrolidine (11b). Synthesized according to method G using compound **3b** (590 mg, 1.86 mmol). The crude product was obtained, which after flash chromatography (EtOAc/MeOH 99:1 → 23:2) yielded **11b** as a pale yellow sap (250 mg, 45 %). ^{1}H NMR δ 7.24 – 7.06 (m, 5H), 6.45 – 6.23 (m, 1H), 4.68 – 4.56 (m, 2H), 3.67 – 3.49 (m, 2H), 2.95 – 2.79 (m, 2H), 2.52 – 2.37 (m, 2H), 2.27 – 2.01 (m, 4H), 1.24 (d, *J* = 6.9 Hz, 3H). ^{13}C NMR δ 171.88, 171.71, 140.72, 128.62, 128.43, 126.36, 118.20, 46.60, 46.58, 46.48, 38.11, 31.59, 29.88, 25.33, 18.09.

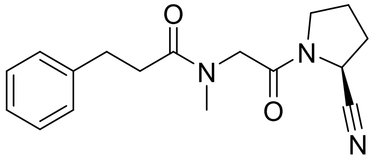

N-(3-Phenylpropanoyl)-sarcosinyl-2(S)-cyanopyrrolidine (11c). Synthesized according to method G using compound **3c** (591 mg, 1.86 mmol). The crude product was obtained, which after flash chromatography (EtOAc → EtOAc/MeOH 9:1) yielded **11c** (300 mg, 54 %). ^{1}H NMR δ 7.41 – 7.10 (m, 5H), 4.97 (dd, J = 7.7, 2.0 Hz, 0.2H), 4.79 – 4.74 (m, 0.8H), 4.54 – 4.43 (m, 1H), 4.01 – 3.94 (m, 0.2H), 3.85 – 3.76 (m, 0.8H), 3.69 – 3.49 (m, 2H), 3.13 (s, 0.6H), 3.12 (s, 2.4H), 3.02 – 2.95 (m, 2H), 2.76 – 2.66 (m, 2H), 2.40 – 2.06 (m, 4H) (two rotamers 4:1). ^{13}C NMR δ 173.11, 167.51, 141.29, 128.58, 128.47, 126.22, 118.37, 49.93, 46.58, 45.87, 36.96, 35.11, 31.06, 29.88, 25.32. (additional set of signals from minor rotamers (ca 20%) can be seen).

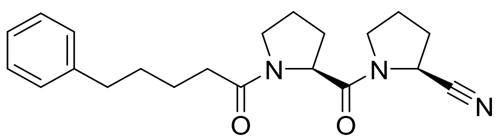

N-(5-Phenylpentanoyl)-L-prolyl-2(S)-cyanopyrrolidine (13a). Synthesized according to method G using compound **5a** (518 mg, 1.39 mmol). The crude product was obtained as a yellow sap, which after flash chromatography (EtOAc → EtOAc/MeOH 19:1) yielded **13a** as a colorless sap (392 mg, 80 %). ^{1}H NMR δ 7.29 – 6.99 (m, 5H), 4.86 – 4.64 (m, 1H), 4.48 (dd, *J* = 8.4, 3.9 Hz, 1H), 3.93 – 3.71 (m, 1H), 3.71 – 3.53 (m, 2H), 3.49 – 3.32 (m, 1H), 2.70 – 2.46 (m, 2H), 2.33 – 1.99 (m, 8H), 1.96 – 1.84 (m, 2H), 1.71 – 1.51 (m, 4H). ^{13}C NMR δ 171.91, 171.51, 142.39, 128.51, 128.37, 125.79, 118.76, 57.44, 47.39, 46.58, 46.45, 35.86, 34.40, 31.20, 29.80, 28.84, 25.48, 25.03, 24.34.

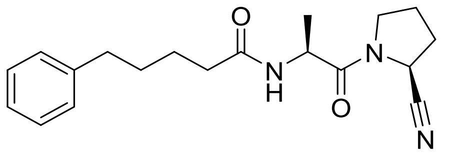

N-(5-Phenylpentanoyl)-L-alanyl-2(S)-cyanopyrrolidine (13b). Synthesized according to method G using compound **5b** (607 mg, 1.76 mmol). The crude product was obtained as a pale yellow oil, which after flash chromatography (EtOAc/MeOH 49:1 → 17:3) yielded **13b** as a colorless sap (255 mg, 44 %). ^{1}H NMR δ 7.33 – 7.11 (m, 5H), 6.44 (d, *J* = 7.7 Hz, 1H), 4.79 – 4.61 (m, 2H), 3.78 – 3.56 (m, 2H), 2.62 (t, *J* = 7.1 Hz, 2H), 2.35 – 2.09 (m, 6H), 1.76 – 1.54 (m, 4H), 1.36 (d, *J* = 6.9 Hz, 3H). ^{13}C NMR δ 172.57, 171.99, 142.24, 128.47, 128.41, 125.86, 118.22, 46.59, 46.56, 46.47, 36.31, 35.73, 31.07, 29.86, 25.32, 25.25, 18.13.

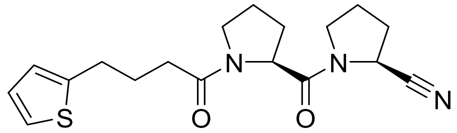

N-(4-(Thiophen-2-yl)butanoyl)-L-prolyl-2(S)-cyanopyrrolidine (14a). Synthesized according to method G using compound **6a** (652 mg, 1.8 mmol). The crude product was obtained, which after flash chromatography (EtOAc/MeOH 49:1 → 17:3) yielded **14a** (487 mg, 79 %). ^{1}H NMR δ 7.15 – 7.08 (m, 1H), 6.96 – 6.88 (m, 1H), 6.83 – 6.79 (m, 1H), 4.87 – 4.78 (m, 1H), 4.57 (dd, *J* = 8.3, 4.0 Hz, 1H), 3.93 – 3.83 (m, 1H), 3.71 – 3.57 (m, 2H), 3.53 – 3.38 (m, 1H), 2.93 – 2.83 (m, 2H), 2.42 – 2.29 (m, 2H), 2.29 – 1.91 (m, 10H). ^{13}C NMR δ 171.40, 171.34, 144.46, 126.76, 124.54, 123.13, 118.65, 57.39, 47.27, 46.51, 46.35, 33.20, 29.71, 29.16, 28.75, 26.34, 25.39, 24.90.

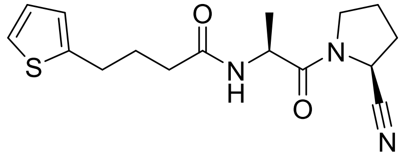

N-(4-(Thiophen-2-yl)butanoyl)-L-alanyl-2(S)-cyanopyrrolidine (14b). Synthesized according to method G using compound **6b** (226 mg, 0.67 mmol). The crude product was obtained as a brown oil, which after flash chromatography (EtOAc → EtOAc/MeOH 19:1) yielded **14b** as an orange sap (143 mg, 67 %). ^{1}H NMR δ 7.13 – 6.95 (m, 1H), 6.93 – 6.82 (m, 1H), 6.80 – 6.66 (m, 1H), 6.43 (d, *J* = 7.8 Hz, 0.3H), 6.35 (d, *J* = 7.7 Hz, 0.55H), 6.20 (d, *J* = 7.9 Hz, 0.15H), 4.89 – 4.52 (m, 2H), 3.91 – 3.30 (m, 2H), 2.91 – 2.70 (m, 2H), 2.36 – 2.04 (m, 6H), 2.00 – 1.86 (m, 2H), 1.37 (d, *J* = 6.9 Hz, 0.4H), 1.30 (d, *J* = 6.9 Hz, 1.7H), 1.25 (d, *J* = 6.8 Hz, 0.9H) (three rotamers 11:6:3). ^{13}C NMR δ 172.14, 171.95, 144.22, 126.92, 124.69, 123.35, 118.23, 46.62, 46.62, 46.50, 35.35, 29.89, 29.23, 27.40, 25.35, 18.16 (two additional sets of signals from minor rotamers (ca. 30 and 15 %) can be seen).

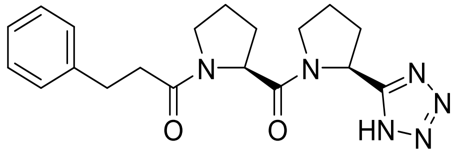

Method H: Synthesis of *N*-(3-phenylpropanoyl)-L-prolyl-2(S)-tetrazolylpyrrolidine (15a). A solution of compound **11a** (741 mg, 2.3 mmol), NH_4Cl (243 mg, 4.5 mmol), and NaN_3 (296 mg, 4.6 mmol) in anhydrous DMF (18 mL) under Ar was heated to 100 °C for 16 h. The liquid fraction was separated and co-evaporated with toluene at 60 °C to obtain the crude product, which after flash chromatography (EtOAc/MeOH 9:1 → 1:1) yielded **15a** as a white foam (263 mg, 31 %). ^{1}H NMR δ 7.30 – 7.00 (m, 5H), 5.37 (dd, J = 8.2, 2.5 Hz, 0.5H), 5.12 (d, J = 7.3 Hz, 0.5H), 4.51 (dd, J = 7.8, 5.0 Hz, 0.5H), 4.35 (dd, J = 7.7, 4.9 Hz, 0.5H), 3.93 – 3.82 (m, 0.5H), 3.79 – 3.69 (m, 0.5H), 3.68 – 3.49 (m, 2H), 3.46 – 3.38 (m, 0.5H), 3.36 – 3.27 (m, 0.5H), 3.04 – 2.85 (m, 1.5H), 2.82 – 2.69 (m, 0.5H), 2.68 – 1.50 (m, 10H) (two rotamers 1:1). ^{13}C NMR δ 172.80 (br), 172.76, 171.65, 169.97, 156.43, 141.00, 140.81, 128.70, 128.58, 128.56, 128.47, 126.43, 126.25, 59.05, 58.16, 53.09, 50.93, 48.22, 47.48, 47.42, 46.59, 36.69, 36.38, 32.38, 30.80, 30.77, 29.39 (br), 29.32, 28.87, 25.62, 25.05 (two peaks), 22.19 (double set of signals due to rotamers). HRMS (ESI-QTOF) m/z: $[M + H]^+$ Calcd for $C_{19}H_{25}N_6O_2$ 369.2039; Found 369.2040.

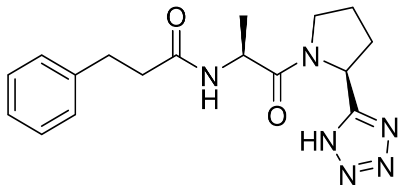

***N*-(3-Phenylpropanoyl)-L-alanyl-2(S)-tetrazolylpyrrolidine (15b).** Synthesized according to method H using compound **11b** (310 mg, 1.04 mmol). The crude product was obtained, which after flash chromatography (EtOAc/MeOH 9:1 → 7:3) yielded **15b** as a brown foam (98 mg, 28 %). ^{1}H NMR (Methanol-d_4) δ 7.31 – 7.11 (m, 5H), 5.54 – 5.49 (m, 0.1H), 5.37 (dd, *J* = 8.2, 3.5 Hz, 0.9H), 4.60 (q, *J* = 7.0 Hz, 0.9H), 4.44 (q, *J* = 6.7 Hz, 0.1H), 3.83 – 3.70 (m, 1.8H), 3.70 – 3.51 (m, 0.2H), 2.94 – 2.79 (m, 2H), 2.55 – 2.49 (m, 1.8H), 2.49 – 2.41 (m, 0.2H), 2.41 – 2.30 (m, 1.2H), 2.26 – 2.00 (m, 2.8H), 1.29 (d, J = 6.9 Hz, 0.3H), 1.24 (d, J = 6.9 Hz, 2.7H) (two rotamers 9:1). ^{13}C NMR (Methanol-d_4) δ 174.83, 173.72, 159.87, 142.12, 129.44, 129.39, 127.20, 53.09, 48.37, 48.18, 38.26, 32.62, 32.04, 25.79, 16.69 (additional set of signals from minor rotamer (ca. 10 %) can be seen). HRMS (ESI-QTOF) m/z: $[M + H]^+$ Calcd for $C_{17}H_{23}N_6O_2$ 343.1882; Found 343.1884.

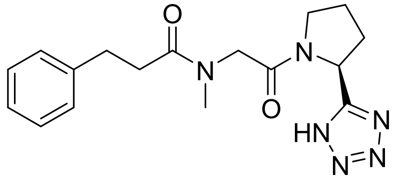

***N*-(3-Phenylpropanoyl)-sarcosinyl-2(S)-tetrazolylpyrrolidine (15c).** Synthesized according to method H using compound **11c** (300 mg, 1.00 mmol). The crude product was obtained, which after flash chromatography (EtOAc/MeOH 23:2 → 3:2) yielded **15c** as a brown foam (166 mg, 48 %). ^{1}H NMR (Methanol-d4) δ 7.30 – 7.07 (m, 5H), 5.60 – 5.40 (m, 1H), 4.41 – 4.12 (m, 2H), 3.84 – 3.53 (m, 2H), 3.08 – 2.52 (m, 7H), 2.51 – 2.03 (m, 4H). ^{13}C NMR (Methanol-d4) δ 174.35, 168.42, 141.03, 128.07, 128.04, 125.74, 52.01, 49.98, 46.08, 36.10, 34.51, 30.76, 30.66, 24.24. $[M + H]^+$ Calcd for $C_{17}H_{23}N_6O_2$ 343.1882; Found 343.1881.

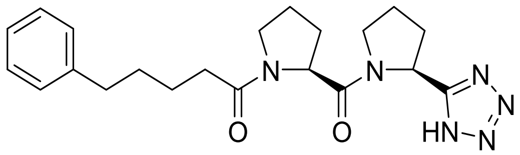

N-(5-Phenylpentanoyl)-L-prolyl-2(S)-tetrazolylpyrrolidine (16a). Synthesized according to method H using compound **13a** (376 mg, 1.1 mmol). The crude product as obtained as a brown oil, which after flash chromatography (EtOAc/MeOH 19:1 → 4:1) yielded **16a** as an orange oil (278 mg, 66 %). ^{1}H NMR δ 7.36 – 7.03 (m, 5H), 5.42 – 5.30 (m, 0.5H), 5.17 (d, *J* = 7.3 Hz, 0.5H), 4.56 (dd, *J* = 7.9, 4.9 Hz, 0.5H), 4.42 (dd, *J* = 7.5, 4.7 Hz, 0.5H), 3.97 – 3.37 (m, 4H), 3.07 (dd, *J* = 12.6, 6.3 Hz, 0.5H), 2.66 (t, *J* = 7.2 Hz, 1H), 2.60 – 2.48 (m, 1.5H), 2.48 – 2.31 (m, 2H), 2.30 – 1.86 (m, 6H), 1.84 – 1.60 (m, 3H), 1.58 – 1.33 (m, 2H) (two rotamers 1:1). ^{13}C NMR δ 173.53, 172.92, 172.25, 169.98, 156.45, 142.28, 142.23, 128.52, 128.51, 128.44, 128.36, 125.88, 125.78, 58.99, 58.01, 53.05, 50.82, 48.25, 47.47, 47.38, 46.56, 35.85, 35.76, 34.56, 34.12, 32.37, 31.05, 31.04, 29.32, 29.29, 28.85, 25.62, 25.09, 25.07, 24.24, 24.14, 22.18 (double set of signals due to rotamers). HRMS (ESI-QTOF) m/z: [M + H]$^+$ Calcd for $C_{21}H_{29}N_6O_2$ 397.2352; Found 397.2355.

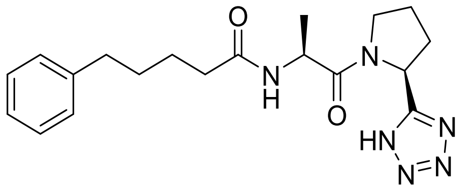

N-(5-Phenylpentanoyl)-L-alanyl-2(S)-tetrazolylpyrrolidine (16b). Synthesized according to method H using compound **13b** (255 mg, 0.78 mmol). The crude product was obtained, which after flash chromatography (EtOAc/MeOH/AcOH 25:74.9:0.1) yielded **16b** as a brown foam (56 mg, 19 %). ^{1}H NMR (Methanol-d_4) δ 7.32 – 6.99 (m, 5H), 5.52 – 5.47 (m, 0.1H), 5.47 – 5.32 (m, 0.9H), 4.61 (q, *J* = 7.0 Hz, 0.9H), 4.44 (q, *J* = 6.9 Hz, 0.1H), 3.94 – 3.74 (m, 1.8H), 3.73 – 3.46 (m, 0.2H), 2.69 – 2.51 (m, 2H), 2.47 – 1.81 (m, 6H), 1.70 – 1.51 (m, 4H), 1.33 (d, J = 6.9 Hz, 0.3H), 1.27 (d, *J* = 6.9 Hz, 1.7H) (two rotamers 9:1). ^{13}C NMR (Methanol-d_4) δ 175.77, 173.86, 160.81, 143.52, 129.41, 129.30, 126.74, 53.33, 48.40, 48.27, 36.55, 36.34, 32.18, 32.04 (br), 26.42, 25.78, 16.73 (additional set of signals from minor rotamer (ca. 10 %) can be seen). HRMS (ESI-QTOF) m/z: [M + H]$^+$ Calcd for $C_{19}H_{27}N_6O_2$ 371.2195; Found 371.2193.

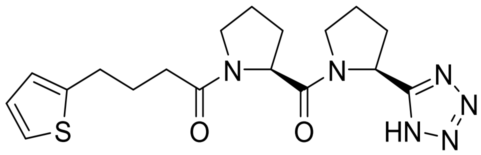

N-(4-(Thiophen-2-yl)butanoyl)-L-prolyl-2(S)-tetrazolylpyrrolidine (17a). Synthesized according to method H using compound **14a** (455 mg, 1.3 mmol). The crude product was obtained, which after flash chromatography (EtOAc/MeOH 9:1 → 1:1) yielded **17a** as a yellow sap (82 mg, 16 %). ^{1}H NMR δ 7.14 (dd, *J* = 5.1, 1.2 Hz, 0.6H), 7.12 (dd, *J* = 5.1, 1.2 Hz, 0.4H), 6.96 – 6.89 (m, 1H), 6.86 (dq, *J* = 3.3, 1.0 Hz, 0.6H), 6.77 (dq, *J* = 3.3, 1.0 Hz, 0.4H), 5.40 (dd, *J* = 8.2, 2.7 Hz, 0.4H), 5.19 (d, *J* = 7.3 Hz, 0.6H), 4.59 (dd, *J* = 7.8, 4.8 Hz, 0.4H), 4.43 (dd, *J* = 7.6, 4.8 Hz, 0.6H), 4.00 – 3.42 (m, 4H), 3.17 – 3.02 (m, 0.6H), 3.00 – 2.88 (m, 1.2H), 2.88 – 2.72 (m, 0.8H), 2.64 – 2.56 (m, 0.4H), 2.55

– 1.60 (m, 11H) (two rotamers 3:2). ^{13}C NMR δ 173.06, 169.82, 156.30, 144.08, 126.83, 124.80, 123.29, 58.92, 52.97, 48.12, 46.48, 33.23, 32.30, 29.22, 29.00, 26.30, 24.97, 22.09 (additional set of signals from minor rotamer (ca. 40 %) can be seen). HRMS (ESI-QTOF) m/z: [M + H]$^+$ Calcd for $C_{18}H_{25}N_6O_2S$ 389.1760; Found 389.1761.

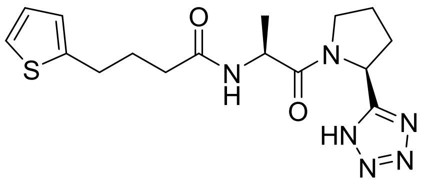

***N*-(4-(Thiophen-2-yl)butanoyl)-L-alanyl-2(S)-tetrazolylpyrrolidine (17b).** Synthesized according to method H using compound **14b** (143 mg, 0.45 mmol). The crude product was obtained as oily brown solids, which after flash chromatography (EtOAc/MeOH 49:1 → 4:1) yielded **17b** as a brown foam (64 mg, 40 %). ^{1}H NMR (Methanol-*d*$_4$) δ 7.23 – 7.12 (m, 1H), 6.96 – 6.86 (m, 1H), 6.86 – 6.75 (m, 1H), 5.45 – 5.35 (m, 1H), 4.68 – 4.53 (m, 1H), 4.04 – 3.61 (m, 2H), 2.92 – 2.80 (m, 2H), 2.57 – 2.33 (m, 1H), 2.33 – 1.87 (m, 7H), 1.33 (d, J = 7.0 Hz, 1.2H), 1.29 (d, J = 7.0 Hz, 1.8H) (two major rotamers 3:2 and additional signals from minor rotamers can be seen). ^{13}C NMR (Methanol-*d*$_4$) δ 174.36, 173.99, 172.62, 172.48, 158.29, 155.90, 144.06, 144.01, 126.33 (two signals), 124.26, 124.20, 122.71 (two signals), 52.29, 51.55, 47.18, 47.05, 46.84, 46.72, 34.19, 34.13, 30.80, 30.65, 28.71, 28.65, 27.53, 27.48, 24.46, 24.12, 15.22, 15.15 (double set of signals due to rotamers). HRMS (ESI-QTOF) m/z: [M + H]$^+$ Calcd for $C_{16}H_{23}N_6O_2S$ 363.1603; Found 363.1603.

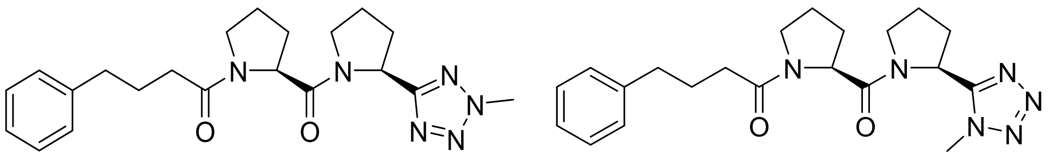

N-(4-Phenylbutanoyl)-L-prolyl-2(S)-(1-methyltetrazolyl)pyrrolidine and N-(4-phenylbutanoyl)-L-prolyl-2(S)-(2-methyltetrazolyl)pyrrolidine (18a and 19a). K_2CO_3 (240 mg, 1.7 mmol) and MeI (0.11 mL, 1.7 mmol) were added to a solution of compound **1a** (332 mg, 0.87 mmol) in anhydrous DMF (15 mL) and the mixture was stirred at room temperature for 23 h. The solution was diluted with EtOAc, washed with a saturated solution of $NaHCO_3$ and brine, dried over anhydrous Na_2SO_4, filtered, and evaporated to provide the crude product as a pale yellow oil, which after flash chromatography (EtOAc → EtOAc/MeOH 4:1) yielded **18a** (49 mg, 14 %) and **19a** (129 mg, 38 %), both as pale yellow saps. **18a**: ^{1}H NMR δ 7.31 – 6.99 (m, 5H), 5.18 (dd, *J* = 8.1, 3.5 Hz, 1H), 4.52 (dd, *J* = 8.3, 4.3 Hz, 1H), 4.09 (s, 3H), 3.99 – 3.88 (m, 1H), 3.77 – 3.66 (m, 1H), 3.49 – 3.28 (m, 2H), 2.60 (t, *J* = 7.5 Hz, 2H), 2.57 – 2.48 (m, 1H), 2.34 – 1.62 (m, 11H) (additional set of signals from minor rotamer (ca. 5 %) and other product (ca. 5 %) can be seen). ^{13}C NMR δ 171.71, 171.43, 156.43, 141.80, 128.62, 128.42, 125.98, 57.72, 49.67, 47.36, 46.95, 35.25, 34.02, 33.59, 30.79, 28.57, 26.03, 25.39, 24.84 (additional set of signals from minor rotamer and other product can be seen). **19a**: ^{1}H NMR δ 7.26 – 7.01 (m, 5H), 5.44 (dd, *J* = 8.0, 2.8 Hz, 1H), 4.64 (dd, *J* = 7.9, 3.5 Hz, 1H), 4.20 (s, 3H), 3.96 – 3.87 (m, 1H), 3.76 – 3.67 (m, 1H), 3.53 – 3.26 (m, 2H), 2.60 (t, *J* = 7.5 Hz, 2H), 2.32 – 1.67 (m, 12H) (additional set of signals from minor rotamer (ca. 5 %) and other product (ca. 10 %) can be seen). ^{13}C NMR δ 171.58, 170.98, 168.09, 141.91, 128.62,

128.37, 125.87, 57.74, 52.79, 47.28, 46.85, 39.42, 35.28, 33.62, 31.38, 28.51, 26.03, 24.70, 24.62 (additional set of signals from minor rotamer and other product can be seen). HRMS (ESI-QTOF) m/z: [M + H]$^+$ Calcd for $C_{21}H_{29}N_6O_2$ 397.2352; Found 397.2351 and 397.2353 for **18a** and **19a**, respectively.

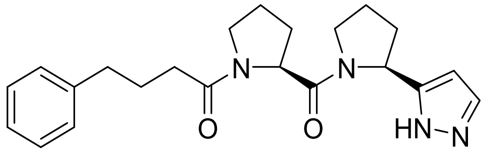

Method I: Synthesis of N-(4-phenylbutanoyl)-L-prolyl-2(S)-(pyrazol-3-yl)pyrrolidine (30a). Compound **10a** (122 mg, 0.34 mmol) was dissolved in DMF-DMA (0.41 mL, 3.1 mmol) and refluxed for 2 h. The solution was cooled to room temperature, diluted with hexane, and the solvents evaporated to provide the intermediate as a yellow oil (134 mg, 95 %), which was used immediately without further purification. The intermediate (134 mg, 0.33 mmol) in AcOH (0.2 mL) was added to N_2H_2 monohydrate (0.02 mL, 0.38 mmol) in AcOH (0.35 mL). The resulting mixture was stirred at 90 °C for 1 h then poured into H_2O and extracted with DCM. The organic phase was washed with a saturated solution of $NaHCO_3$, dried over anhydrous Na_2SO_4, filtered, and evaporated to provide the crude product as an off white foam, which after flash chromatography (EtOAc/MeOH 19:1 → 7:3) yielded **30a** as a colorless oil (31 mg, 25 %). ^{1}H NMR δ 7.54 (d, J = 1.8 Hz, 0.4H), 7.43 (d, J = 1.8 Hz, 0.6H), 7.31 – 7.07 (m, 6H), 6.14 – 6.11 (m, 0.4H), 6.10 – 6.06 (m, 0.6H), 5.35 – 5.26 (m, 0.6H), 5.07 – 5.01 (m, 0.4H), 4.65 – 4.60 (m, 0.6H), 4.56 – 4.47 (m, 0.4H), 3.98 – 3.26 (m, 4H), 2.77 – 2.56 (m, 2H), 2.46 – 1.66 (m, 12H) (two rotamers 3:2) (additional signals from minor rotamers). ^{13}C NMR δ 172.74, 172.43, 171.70, 170.53, 146.34, 141.84, 137.88 – 136.56 (m), 128.74, 128.66, 128.44, 128.41, 125.98, 101.93, 101.73, 58.78, 57.69, 54.49, 52.64, 47.77, 47.41, 47.26, 46.07, 35.30, 35.22, 33.85, 33.69, 33.68, 33.67, 29.19, 29.01, 26.08, 26.03, 25.40, 25.07, 24.99, 22.14 (double set of signals due to rotamers). HRMS (ESI-QTOF) m/z: [M + H]$^+$ Calcd for $C_{22}H_{29}N_4O_2$ 381.2291; Found 381.2292.

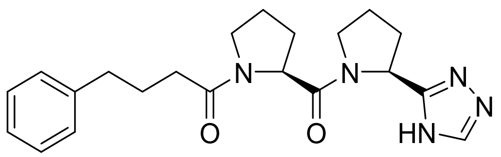

***N*-(4-Phenylbutanoyl)-L-prolyl-2(S)-(1,2,4-triazolyl)pyrrolidine (20a).** Synthesized according to method I using **4a** (317 mg, 0.89 mmol). The intermediate was obtained as a yellow oil (quantitative) and the crude product as a white foam, which after flash chromatography (EtOAc/MeOH 49:1 → 4:1) yielded **20a** as a white foam (260 mg, 77 %). ^{1}H NMR δ 7.95 (s, 0.6H), 7.85 (s, 0.4H), 7.33 – 7.08 (m, 5H), 5.33 – 5.21 (m, 0.4H), 5.04 (d, *J* = 7.2 Hz, 0.6H), 4.62 (dd, *J* = 8.1, 4.5 Hz, 0.4H), 4.42 (dd, *J* = 7.6, 5.2 Hz, 0.6H), 3.99 – 3.83 (m, 0.4H), 3.73 – 3.39 (m, 3.6H), 2.99 – 2.85 (m, 0.6H), 2.77 – 2.62 (m, 2H), 2.61 – 2.53 (m, 0.4H), 2.49 – 1.57 (m, 11H) (two rotamers 3:2). ^{13}C NMR δ 172.87, 172.71, 171.79, 170.32, 157.82, 156.16, 141.77, 141.64, 128.70, 128.61, 128.45, 128.43, 126.01, 125.98, 58.87, 57.89, 54.98, 53.00, 47.96, 47.48, 47.41, 46.57, 35.25, 35.14, 33.64, 33.59, 32.64, 29.19, 28.86, 28.52, 26.07, 26.01, 25.41, 25.12, 25.01, 22.19 (double set of signals due to rotamers). HRMS (ESI-QTOF) m/z: [M + H]$^+$ Calcd for $C_{21}H_{28}N_5O_2$ 382.2243; Found 382.2245.

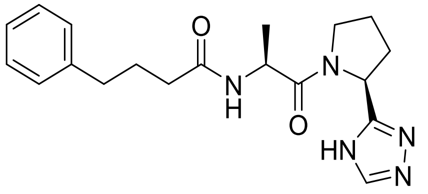

N-(4-Phenylbutanoyl)-L-alanyl-2(S)-(1,2,4-triazolyl)pyrrolidine (20b). Synthesized according to method I using **4b** (60 mg, 0.18 mmol). The intermediate was obtained as an orange oil (quantitative) and the crude product as a white foam, which after flash chromatography (EtOAc/MeOH 93:7 → 4:1) yielded **20b** as a white foam (43 mg, 67 %). ^{1}H NMR δ 13.09 (s, 1H), 8.14 – 8.02 (m, 0.5H), 7.82 (s, 0.5H), 7.74 (s, 0.5H), 7.31 – 7.08 (m, 5H), 7.08 – 7.00 (m, 0.5H), 5.43 – 5.26 (m, 1H), 4.92 – 4.78 (m, 0.5H), 4.70 (p, *J* = 6.9 Hz, 0.5H), 4.04 – 3.42 (m, 2H), 2.86 – 2.70 (m, 0.5H), 2.70 – 2.49 (m, 2.5H), 2.47 – 1.80 (m, 8H), 1.36 (d, *J* = 7.0 Hz, 1.5H), 1.21 (d, *J* = 7.0 Hz, 1.5H) (two rotamers 1:1) (additional signals from minor rotamers can be seen). ^{13}C NMR δ 173.90, 173.67, 173.11, 172.76, 156.54, 150.58, 149.63, 141.70, 141.51, 128.63, 128.59, 128.47, 126.04, 53.92, 52.55, 47.54, 47.39, 47.17, 46.32, 35.53, 35.41, 35.39, 35.20, 29.90, 28.18, 27.20, 27.05, 25.43, 24.67, 17.97, 17.30 (double set of signals due to rotamers). HRMS (ESI-QTOF) m/z: [M + H]$^+$ Calcd for $C_{19}H_{26}N_5O_2$ 356.2087; Found 356.2089.

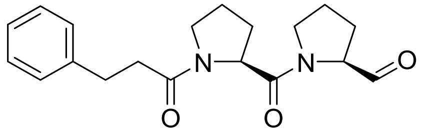

Method J: Synthesis of *N*-(3-phenylpropanoyl)-L-prolyl-L-prolinal (21a). NaBr (85 mg, 0.83 mmol) and TEMPO (6 mg, 0.04 mmol) were added to a solution of compound **7a** (1.37 g, 4.1 mmol) in DCM (10 mL). Aqueous NaOCl (3.3 mL, 10 % (w/V), 5.4 mmol) and aqueous $NaHCO_3$ (3.5 mL, 5 % (w/V), 2.1 mmol) were added at 0 °C and stirring was continued for 20 min before filtering the resulting suspension through Celite. The aqueous phase was extracted with DCM and the combined organic phases dried over anhydrous Na_2SO_4, filtered, and evaporated to provide the crude product as an orange sap, which after flash chromatography (EtOAc/MeOH 99:1 → 9:1) yielded **21a** as a colorless sap (1.10 g, 81 %). Flash chromatography with MeOH resulted in partial hemiacetal formation, which was reversed back to the aldehyde in vacuum. ^{1}H NMR δ 9.52 (d, *J* = 1.4 Hz, 1H), 7.44 – 6.98 (m, 5H), 4.76 – 4.64 (m, 1H), 4.64 – 4.55 (m, 1H), 4.00 – 3.90 (m, 1H), 3.68 – 3.55 (m, 2H), 3.45 – 3.38 (m, 1H), 3.05 – 2.89 (m, 2H), 2.73 – 2.50 (m, 2H), 2.30 – 1.82 (m, 8H). ^{13}C NMR δ 199.01, 171.75, 171.06, 141.47, 128.60, 128.49, 126.20, 64.89, 57.59, 47.36, 47.09, 36.61, 30.90, 29.19, 25.56, 25.09, 24.91.

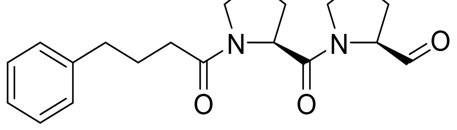

N-(4-Phenylbutanoyl)-L-prolyl-L-prolinal (22a). Synthesized according to method J using compound **8a** (1.00 g, 2.9 mmol). The crude product was obtained as an orange sap, which after flash chromatography (EtOAc/MeOH 99:1 → 9:1) yielded **22a** as a colorless sap (820 mg, 82 %). ^{1}H NMR δ 9.52 (d, J = 1.4 Hz, 1H), 7.42 – 7.10 (m, 5H), 4.73 – 4.63 (m, 1H), 4.63 – 4.56 (m, 1H),

3.98 – 3.89 (m, 1H), 3.65 – 3.54 (m, 2H), 3.51 – 3.41 (m, 1H), 2.73 – 2.59 (m, 2H), 2.39 – 1.82 (m, 12H). ^{13}C NMR δ 199.08, 171.80, 171.66, 141.86, 128.65, 128.42, 125.94, 64.86, 57.52, 47.34, 47.07, 35.27, 33.61, 29.20, 26.05, 25.55, 25.08, 24.92.

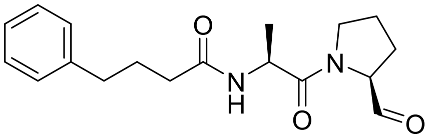

N-(4-Phenylbutanoyl)-L-alanyl-L-prolinal (22b). Synthesized according to method J using compound **8b** (577 mg, 1.8 mmol). The crude product was obtained, which after flash chromatography (EtOAc/MeOH 99:1 → 23:2) yielded **22b** (439 mg, 77 %). Due to insufficient vacuum, this compound was still partially in hemiacetal form (25 %). ^{1}H NMR δ 9.54 – 9.41 (m, 1H), 7.35 – 7.09 (m, 5H), 6.50 – 6.40 (m, 0.75H), 6.38 – 6.33 (m, 0.25H), 4.83 – 4.70 (m, 0.75H), 4.61 – 4.43 (m, 0.75H), 4.40 – 4.28 (m, 0.25H), 4.28 – 4.16 (m, 0.25H), 3.80 – 3.44 (m, 2H), 2.72 – 2.58 (m, 2H), 2.49 – 2.14 (m, 2H), 2.14 – 1.75 (m, 6H), 1.60 – 1.32 (m, 3H) (additional signals from remaining hemiacetal can be seen). ^{13}C NMR δ 198.27, 172.27, 172.25, 141.54, 128.60, 128.50, 126.07, 64.95, 47.19, 46.60, 35.84, 35.30, 27.16, 25.67, 24.98, 18.70 (additional signals remaining hemiacetal can be seen).

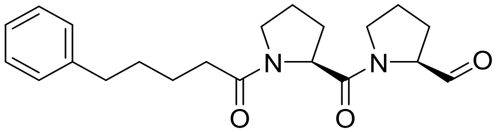

***N*-(5-Phenylpentanoyl)-L-prolyl-L-prolinal (23a).** Synthesized according to method J using compound **9a** (2.66 g, 7.4 mmol). The crude product was obtained as an orange oil, which after flash chromatography (EtOAc/MeOH 99:1 → 9:1) yielded **23a** as a colorless oil (2.15 g, 81 %). ^{1}H NMR δ 9.52 (d, J = 1.4 Hz, 1H), 7.34 – 7.10 (m, 5H), 4.71 – 4.63 (m, 1H), 4.62 – 4.53 (m, 1H), 3.99 – 3.89 (m, 1H), 3.70 – 3.54 (m, 2H), 3.52 – 3.44 (m, 1H), 2.69 – 2.57 (m, 2H), 2.40 – 1.81 (m, 10H), 1.76 – 1.58 (m, 4H). ^{13}C NMR δ 199.09, 171.85, 142.49, 128.54, 128.38, 125.79, 64.87, 57.51, 47.40, 47.08, 35.91, 34.45, 31.28, 29.21, 25.56, 25.09, 24.96, 24.38.

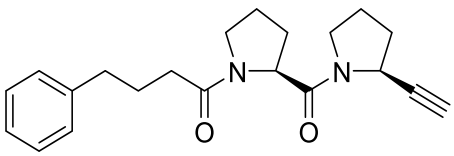

***N*-(4-Phenylbutanoyl)-L-prolyl-2(S)-ethynylpyrrolidine (24a).** Dimethyl 2-oxopropylphosphonate (0.64 mL, 4.6 mmol) was added to a solution of *p*-ABSA (1.2 g, 5.0 mmol) and K_2CO_3 (2.4 g, 17 mmol) in anhydrous MeCN (30 mL) under Ar. The mixture was stirred at room temperature for 2 h before adding a solution of compound **22a** (1.3 g, 3.8 mmol) in anhydrous MeOH (30 mL). Stirring was continued at room temperature for another 19 h. Et_2O was added, the resulting suspension filtered, and the filtrate evaporated. The residue was dissolved in H_2O and extracted with Et_2O and EtOAc. The combined organic phase was dried over anhydrous Na_2SO_4, filtered, and evaporated to provide the crude product as a yellow sap, which after flash chromatography (heptane/EtOAc 1:1 → EtOAc/MeOH 19:1) yielded **24a** (846 mg, 65

%). ^{1}H NMR δ 7.34 – 7.07 (m, 5H), 5.12 – 5.07 (m, 0.05H), 5.05 – 4.99 (m, 0.1H), 4.85 – 4.79 (m, 0.7H), 4.75 – 4.69 (m, 0.1H), 4.64 – 4.56 (m, 0.7H), 4.56 – 4.52 (m, 0.05H), 4.50 – 4.46 (m, 0.1H), 4.46 – 4.40 (m, 0.1H), 3.89 – 3.33 (m, 4H), 2.76 – 2.55 (m, 2H), 2.43 – 1.74 (m, 13H) (four rotamers 14:2:2:1). ^{13}C NMR δ 171.58, 170.67, 141.89, 128.64, 128.38, 125.89, 83.73, 69.78, 57.54, 47.62, 47.34, 46.25, 35.25, 33.61, 31.94, 28.69, 26.05, 25.17, 24.87 (additional signals from minor rotamers can be seen).

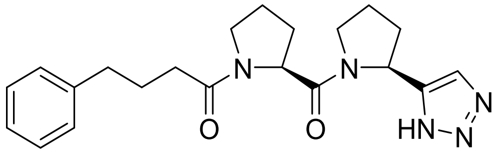

***N*-(4-Phenylbutanoyl)-L-prolyl-2(S)-(1,2,3-triazolyl)pyrrolidine (25a).** A solution of compound **24a** (185 mg, 0.55 mmol) in anhydrous DMF (2 mL) was added to CuI (5 mg, 0.03 mmol) under Ar. Anhydrous MeOH (0.22 mL) and $TMSN_3$ (0.11 mL, 0.82 mmol) were added and the mixture was heated to 100 °C for 19 h. The solution was cooled to room temperature, filtered through Celite, and co-evaporated with MeOH and toluene. The crude product was obtained as brown solids, which after flash chromatography (EtOAc → EtOAc/MeOH 4:1) yielded **25a** as a yellow amorphous solid (129 mg, 62 %). ^{1}H NMR δ 7.40 (s, 0.4H), 7.22 – 7.03 (m, 5H), 5.35 – 5.14 (m, 0.6H), 5.06 (d, J = 7.2 Hz, 0.4H), 4.58 (dd, J = 7.8, 4.0 Hz, 0.6H), 4.53 (br s, 0.4H), 4.45 – 4.36 (m, 0.4H), 3.91 – 3.76 (m, 0.6H), 3.74 – 3.19 (m, 3.4H), 2.85 (s, 0.6H), 2.68 – 2.49 (m, 2H), 2.39 – 1.56 (m, 12H) (two rotamers 3:2). ^{13}C NMR δ 171.88, 171.64 (br), 159.33, 141.84, 128.62, 128.39, 125.91, 57.88, 52.56 (br), 47.40, 47.11, 35.24, 33.62, 30.55 (br), 28.80, 26.05, 24.89, 21.78 (br) (additional set of signals from minor rotamer (ca. 40 %) can be seen). HRMS (ESI-QTOF) m/z: [M + H]$^+$ Calcd for $C_{21}H_{28}N_5O_2$ 382.2243; Found 382.243.

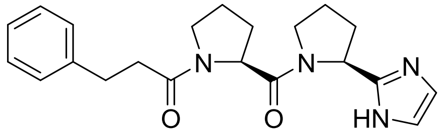

Method K: Synthesis of *N*-(3-phenylpropanoyl)-L-prolyl-2(S)-(imidazol-2-yl)pyrrolidine (26a). A solution of NH_3 (7 N in MeOH, 1.2 mL, 8.3 mmol) in MeOH (1.5 mL) was added to compound **21a** (1.0 g, 3.2 mmol) and the mixture was stirred at room temperature for 30 min. Glyoxal (40 % in H_2O, 0.47 mL, 4.1 mmol) was added slowly dropwise, and stirring was continued for 19 h before removal of MeOH through evaporation. DCM was added and the organic phase was washed with H_2O, dried over anhydrous Na_2SO_4, filtered, and evaporated to provide the crude product, which after flash chromatography (EtOAc/MeOH 19:1 → 3:2) yielded **26a** as an off-white foam (377 mg, 33 %). ^{1}H NMR δ 12.34 (s, 0.7H), 10.42 (s, 0.3H), 7.36 – 7.11 (m, 5H), 7.04 (s, 1.4H), 6.93 (s, 0.6H), 5.26 (dd, *J* = 8.1, 2.6 Hz, 0.3H), 5.00 (d, *J* = 7.1 Hz, 0.7H), 4.61 (dd, *J* = 8.1, 4.8 Hz, 0.3H), 4.48 (dd, *J* = 7.6, 5.1 Hz, 0.7H), 4.15 – 3.37 (m, 4H), 3.13 – 2.83 (m, 3H), 2.78 – 2.49 (m, 2H), 2.40 – 1.53 (m, 7H) (two rotamers 7:3). ^{13}C NMR δ 172.00, 170.29, 146.89, 141.13, 128.62, 128.52, 126.34, 117.27, 58.93, 56.28, 48.04, 46.40, 36.72, 32.55, 30.96, 29.16, 25.04, 22.26 (additional set of signals from minor rotamer (ca. 30 %) can be seen). HRMS (ESI-QTOF) m/z: [M + H]$^+$ Calcd for $C_{21}H_{27}N_4O_2$ 367.2134; Found 367.2130.

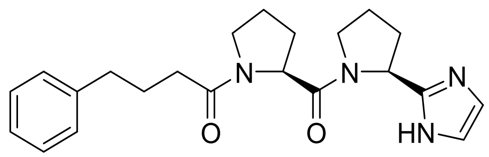

***N*-(4-Phenylbutanoyl)-L-prolyl-2(S)-(imidazol-2-yl)pyrrolidine (27a).** Synthesized according to method K using compound **22a** (720 mg, 2.1 mmol). The crude product was obtained as a yellow foam, which after flash chromatography (EtOAc/MeOH 19:1 → 3:2) yielded **27a** as an off-white foam (137 mg, 17 %). ^{1}H NMR δ 12.38 (s, 0.7H), 10.43 (s, 0.3H), 7.33 – 7.10 (m, 6H), 7.04 (s, 1.4H), 6.93 (s, 0.6H), 5.25 (dd, *J* = 8.1, 2.6 Hz, 0.3H), 5.00 (d, *J* = 7.1 Hz, 0.7H), 4.61 (dd, *J* = 8.1, 4.7 Hz, 0.3H), 4.47 (dd, *J* = 7.7, 4.8 Hz, 0.7H), 3.95 – 3.40 (m, 4H), 3.03 – 2.83 (m, 1H), 2.75 – 2.59 (m, 2H), 2.48 – 1.61 (m, 11H) (two rotamers 7:3). ^{13}C NMR δ 172.67, 170.34, 146.94, 141.68, 128.67, 128.49, 126.08, 117.31, 58.89, 56.23, 48.03, 46.41, 35.28, 33.85, 32.59, 29.18, 26.30, 25.07, 22.27 (additional set of signals from minor rotamer (ca. 30 %) can be seen). HRMS (ESI-QTOF) m/z: [M + H]$^+$ Calcd for $C_{22}H_{29}N_4O_2$ 381.2291; Found 381.2292.

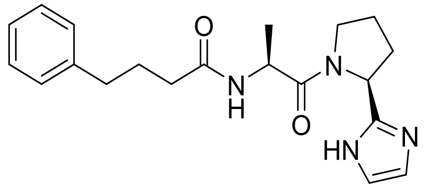

N-(4-Phenylbutanoyl)-L-alanyl-2(S)-(imidazol-2-yl)pyrrolidine (27b). Synthesized according to method K using compound **22b** (439 mg, 1.39 mmol). The crude product was obtained, which after flash chromatography, first using a regular silica column (EtOAc/MeOH 9:1 → 7:3) and then an amine functionalized column ($CHCl_3$ → EtOH/$CHCl_3$ 19:1), yielded **27b** as a white foam (22 mg, 4 %). ^{1}H NMR δ 12.19 (s, 0.3H), 10.31 (s, 0.7H), 7.34 – 7.11 (m, 5H), 7.05 (s, 0.6H), 6.95 (s, 1.4H), 6.50 (d, J = 6.2 Hz, 0.3H), 6.35 (d, J = 7.5 Hz, 0.7H), 5.28 – 5.13 (m, 0.7H), 5.01 (d, J = 7.0 Hz, 0.3H), 4.74 (p, J = 7.0 Hz, 0.7H), 4.50 – 4.37 (m, 0.3H), 3.81 – 3.40 (m, 2H), 3.09 – 2.86 (m, 1H), 2.73 – 2.56 (m, 2H), 2.39 – 2.27 (m, 0.7H), 2.26 – 2.18 (m, 2H), 2.18 – 2.04 (m, 2H), 2.04 – 1.90 (m, 2H), 1.89 – 1.73 (m, 0.3H), 1.37 (d, J = 7.0 Hz, 0.9H), 1.26 (d, J = 6.9 Hz, 2.1H) (two rotamers 7:3). ^{13}C NMR δ 173.21, 172.16, 147.65, 141.51, 128.60, 128.51, 126.10, 116.54, 54.52, 47.60, 46.68, 35.83, 35.30, 27.44, 27.13, 25.43, 18.44 (additional set of signals from minor rotamer (ca. 30 %) can be seen). HRMS (ESI-QTOF) m/z: [M + H]+ Calcd for $C_{20}H_{27}N_4O_2$ 355.2134; Found 355.2134.

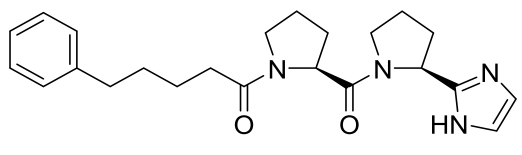

***N*-(5-Phenylpentanoyl)-L-prolyl-2(S)-(imidazol-2-yl)pyrrolidine (28a).** Synthesized according to method K using compound **23a** (2.0 g, 5.6 mmol). The crude product was obtained as a yellow foam, which after flash chromatography (EtOAc/MeOH 19:1 → 3:2) yielded **28a** as an off-white foam (822 mg, 37 %). ^{1}H NMR δ 12.32 (s, 0.7H), 10.42 (s, 0.3H), 7.32 – 7.09 (m, 5H), 7.02 (s, 1.4H), 6.91 (s, 0.6H), 5.23 (dd, *J* = 8.1, 2.6 Hz, 0.3H), 4.98 (d, *J* = 7.1 Hz, 0.7H), 4.59 (dd, *J* = 8.1, 4.7 Hz, 0.3H), 4.48 (dd, *J* = 7.7, 4.9 Hz, 0.7H), 3.93 – 3.47 (m, 4H), 3.02 – 2.83 (m, 1H), 2.73 – 2.53 (m, 2H), 2.48 – 2.00 (m, 6H), 1.98 – 1.87 (m, 2H), 1.82 – 1.58 (m, 5H) (two rotamers 7:3). ^{13}C NMR δ

172.81, 170.29, 146.89, 142.31, 128.49, 128.40, 125.84, 117.27, 58.86, 56.22, 48.08, 46.36, 35.77, 34.69, 32.50, 31.14, 29.16, 25.04, 24.46, 22.24 (additional set of signals from minor rotamer (ca. 30 %) can be seen). HRMS (ESI-QTOF) m/z: $[M + H]^+$ Calcd for $C_{23}H_{31}N_4O_2$ 395.2447; Found 395.2446.

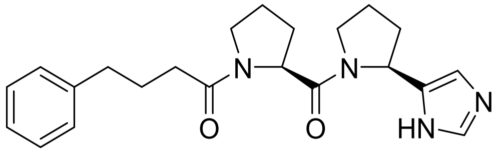

***N*-(4-Phenylbutanoyl)-L-prolyl-2(S)-(imidazol-4-yl)pyrrolidine (29a).** NaCN (7 mg, 0.15 mmol) was added to a suspension of compound **22a** (515 mg, 1.50 mmol) and TosMIC (323 mg, 1.65 mmol) in anhydrous EtOH (8.5 mL) and the mixture was stirred for 2 h at room temperature. EtOH was evaporated, $CHCl_3$ added, the resulting suspension filtered, and the filtrate evaporated. The residue was dissolved in NH_3 (7 N in MeOH, 11 mL) and refluxed for 21 h under Ar before evaporating the solvent. The residue was dissolved in DCM, washed with 1 M Na_2CO_3 and H_2O, dried over anhydrous Na_2SO_4, filtered, and evaporated to provide the crude product as a brown foam, which after flash chromatography (NH functionalized column, EtOAc/MeOH 19:1 → 3:2) yielded **29a** as a brown foam (105 mg, 18 %). ^1H NMR (Methanol-d_4) δ 7.31 – 7.07 (m, 5H), 4.79 – 3.95 (m, 2H), 3.92 – 3.40 (m, 4H), 2.81 – 2.53 (m, 2H), 2.45 – 2.29 (m, 2H), 2.29 – 1.66 (m, 10H) (all peaks were broad). ^{13}C NMR (Methanol-d_4) δ 173.93, 173.89, 143.11, 136.23, 129.54, 129.37, 126.92, 59.49, 48.67, 36.12, 34.42, 27.57, 25.70 (some aliphatic carbons were not found due to broad peaks). HRMS (ESI-QTOF) m/z: $[M + H]^+$ Calcd for $C_{22}H_{29}N_4O_2$ 381.2291; Found 381.2289. Purity 86.3 % according UPLC-MS.

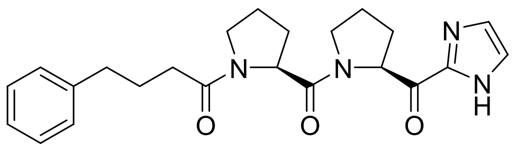

***N*-(4-Phenylbutanoyl)-L-prolyl-2(S)-(imidazole-2-carbonyl)pyrrolidine (2a).** Synthesized according to the previously presented procedure.[2] ^1H NMR δ 11.29 (s, 1H), 7.24 – 7.03 (m, 5H), 7.02 – 6.90 (m, 2H), 5.58 – 5.47 (m, 1H), 4.71 (dd, J = 8.3, 3.1 Hz, 1H), 4.00 – 3.81 (m, 1H), 3.74 – 3.38 (m, 2H), 3.38 – 3.19 (m, 1H), 2.58 (t, J = 7.5 Hz, 2H), 2.40 – 1.69 (m, 12H). ^{13}C NMR δ 188.08, 171.63, 170.94, 143.41, 141.90, 130.99, 128.61, 128.36, 125.87, 120.93, 61.47, 57.79, 47.35, 47.32, 35.22, 33.56, 29.25, 28.55, 26.06, 25.21, 24.58. HRMS (ESI-QTOF) m/z: $[M + H]^+$ Calcd for $C_{23}H_{29}N_4O_3$ 409.2240; Found 409.2240.

Measuring biological activity

Inhibitory Activity. The IC_{50} values for compounds were determined in the microplate assay procedure described in Kilpeläinen et al.[1] Porcine PREP cDNA was expressed in *E.coli* cells and purified as described in Venäläinen et al.[3] The enzyme dilution was preincubated with 0.1 M sodium–potassium phosphate buffer (75 µL, pH 7.0) containing the compounds at desired concentrations in 30 °C for 30 min. The final concentration of the compounds in the assay mixture varied from 1 mM-1 nM and the final concentration of the enzyme was approximately 0.1 nM as measured by Bradford´s method. The enzyme reaction was carried out by adding 25 µL of 4 mM Suc-Gly-Pro-AMC substrate dissolved in 0.1 M sodium–potassium phosphate buffer (pH 7.0) into the assay mixture and incubating at 30° C for 60 min. The reaction was terminated by adding 100 mL of 1 M sodium acetate buffer (pH 4.2). Formation of AMC was measured with Victor[2] multilabel counter (PerkinElmer; excitation/emission 360 nm/ 460 nm), with a standard curve of 0.1-5 nM AMC in 0.1 M sodium–potassium phosphate buffer present. All activity measurements were made at least in triplicate. The inhibitory activities (percent of control) were plotted against the log concentration of the compound, and the IC_{50}-value was determined by non-linear regression utilizing GraphPad Prism 7.0 software.

α-Synuclein Dimerization. αSyn dimerization was assessed by using PCA, which was carried out as described previously in Kilpeläinen et al.[1] In short, N2A cells were seeded on poly-L-lysine-coated 96-well plates (Isoplate™ white wall, PerkinElmer Life Sciences) at the density of 13 000 cells/well. 24 h post-plating, reporter plasmids were transfected with 100 ng of total plasmid DNA per well. N2A cells were transfected with 25 ng of both αSyn-Gluc1 and αSyn-Gluc2 and 50 ng mock-plasmid or non-tagged human PREP expression plasmid 50 ng/well. Lipofectamine 3000 (Thermo Fischer Scientific) was used as the transfection reagent. 100 µL normal growth medium was added to all wells at 24 h post-transfection. 48 hours post-transfection medium was changed to phenol red free DMEM without serum containing the tested compounds at 10 µM concentration, with 0,1% DMSO as vehicle control. Proteasome inhibitors lactacystin (AG Scientific) at 10 µM and MG-132 (Enzo Life Sciences) at 10 µM served as a positive control. The PCA signal was assessed by injecting 25 µL of native coelenterazine (Nanolight Technology) in phenol red free DMEM per well (final concentration 6 µM). The emitted luminescence was read using Varioskan LUX multimode microplate reader (Thermo Scientific). For each experimental condition, 4 replicate wells were used in each experiment, and at least 3 separate experiments for each treatment except for **15c** which had 2 separate experiments.

Autophagic Flux. Autophagic flux was determined by using HEK-293 cells with stable GFP-LC3B-RFP construct expression. Cell line was created according to protocol described in Svarcbahs et al.[4] Autophagic flux was measured as described in Svarcbahs et al.[4] Briefly, GFP-LC3B-RFP expressing HEK-293 cells were seeded at a density of 30 000 cells/well on black poly-L-lysine coated 96-well plates (Costar, Corning). Cells were treated for 24 hours at 10 µM concentrations of test compounds 24 h post-plating, with 0,1 % DMSO as vehicle control. 0.5 µM rapamycin (BML-A275, Enzo Life Sciences) was used as a positive control for autophagy induction and 20 nM bafilomycin 1A (ML1661) as autophagy inhibitor. 24 h after treatment cells were washed once with warm PBS and GFP signal was read with Victor[2] multilabel counter (PerkinElmer; excitation/emission 485nm/535nm). For each experimental condition, 4 replicate wells were

used in each experiment and at least 3 independent experiments were performed. Analysis was performed for only GFP signal as RFP in this construct may be also degraded by lysosomes to certain degree and signal could not be reliably used as a control in well plate format.[5]

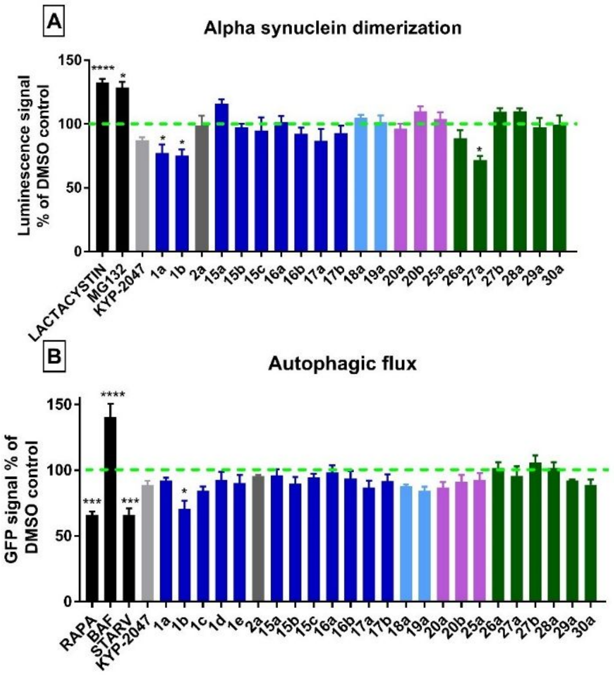

Figure S1. The effect of PREP ligands on (A) αSyn dimerization and (B) autophagic flux. As expected, proteasomal inhibition by lactacystin or MG-132 increased aSyn dimerization 32 % or 29 %, respectively, in the PCA assay compared to DMSO control. Compounds **1a** (23 % decrease), **1b** (25 % decrease) and **27a** (28 % decrease) decreased aSyn dimerization significantly but in the autophagic flux assay, only **1b** (29 % decrease) had a statistically significant effect on autophagic flux. In the autophagy assay, 0.5 µM rapamycin (RAPA; 34 % decrease), 20 nM bafilomycin A1 (BAF; 40 % increase) and amino acid starvation (STARV; 34 % decrease) served as controls. Data is presented as mean+SEM, 1-way ANOVA with Dunnett´s multiple comparison to DMSO control, *p<0.05, *** p<0.001, **** p<0.0001. Hatched green line indicates the level of DMSO control. All compounds were tested at least 3 times except for **15c**, which was tested twice in PCA. The bars are colored for tetrazoles (blue), methyltetrazoles (light blue), triazoles (purple), imidazoles and pyrazole (green).

Molecular docking method

Modelling studies were performed using Schrödinger Maestro.[6] The PREP crystal structure (PDB: 3DDU) was chosen since it is the only structure of human PREP, has a relatively good resolution (1.56 Å), and includes a co-crystalized ligand in the active site.[7] The structure was prepared using Protein Preparation Wizard with mostly default settings.[8] During pre-processing, missing side chains (Gln5, Gln56, Glu65, Glu69, Glu107, Glu163, Gln192, Asp256, Lys335, Gln388, Lys428, Asn483, Ile498, Lys546, Glu624 and Arg664) were filled with Prime[9] and heteroatom states were generated and selected at pH 7.4 with Epik.[10] All water, acetate, and glycerol molecules were removed. Hydrogen bond assignment was done using default settings at pH 7.4 using PROPKA.[11] Minimization was performed using default settings with force field OPLS3e.

Ligands were prepared from their 2D structures with LigPrep using force field OPLS3e.[12] All tautomers and ionization states were generated at pH 7.4 ± 1 with Epik.[10] This resulted in positively charged tetrazoles and both neutral and negatively charged imidazoles. Multiple tautomers for compounds **25a**, **29a**, and **30a** were generated. Because the compounds were synthesized stereoselectively, only S stereoisomers were included.

First glide docking was performed and the grid for that was generated, the center of which was determined based on the co-crystalized ligand. The prepared ligands were docked at XP precision.[13] Afterwards induced fit was also performed, with the docking site determined from the co-crystalized ligand.[14] In both cases the co-crystalized ligand was re-docked to ensure the viability of the model.

The PREP crystal structure with covalently bound KYP-2047 (PDB: 4AN0)[15] was prepared as described above. The protein structures were superimposed using the superposition tool in Schrödinger Maestro.[6] The substructures were defined using C-alpha atoms, as this gave the best results for the binding site residues.

Molecular docking figures

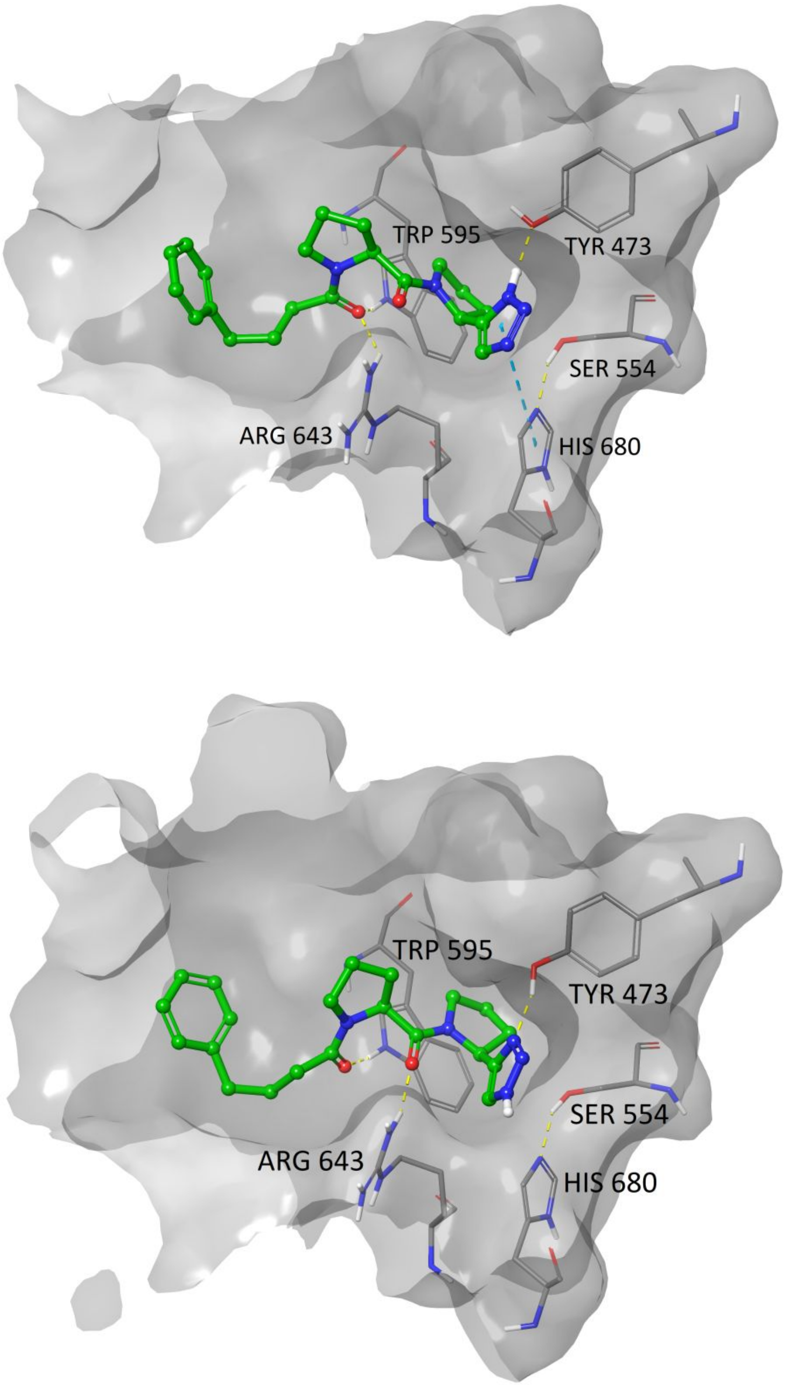

Figure S2. Induced fit docking poses for two tautomers of **25a** at the proteolytic active site of PREP (PDB: 3DDU). Hydrogen bonds are shown as yellow dashed lines and π-π stacking interactions as blue dashed lines.

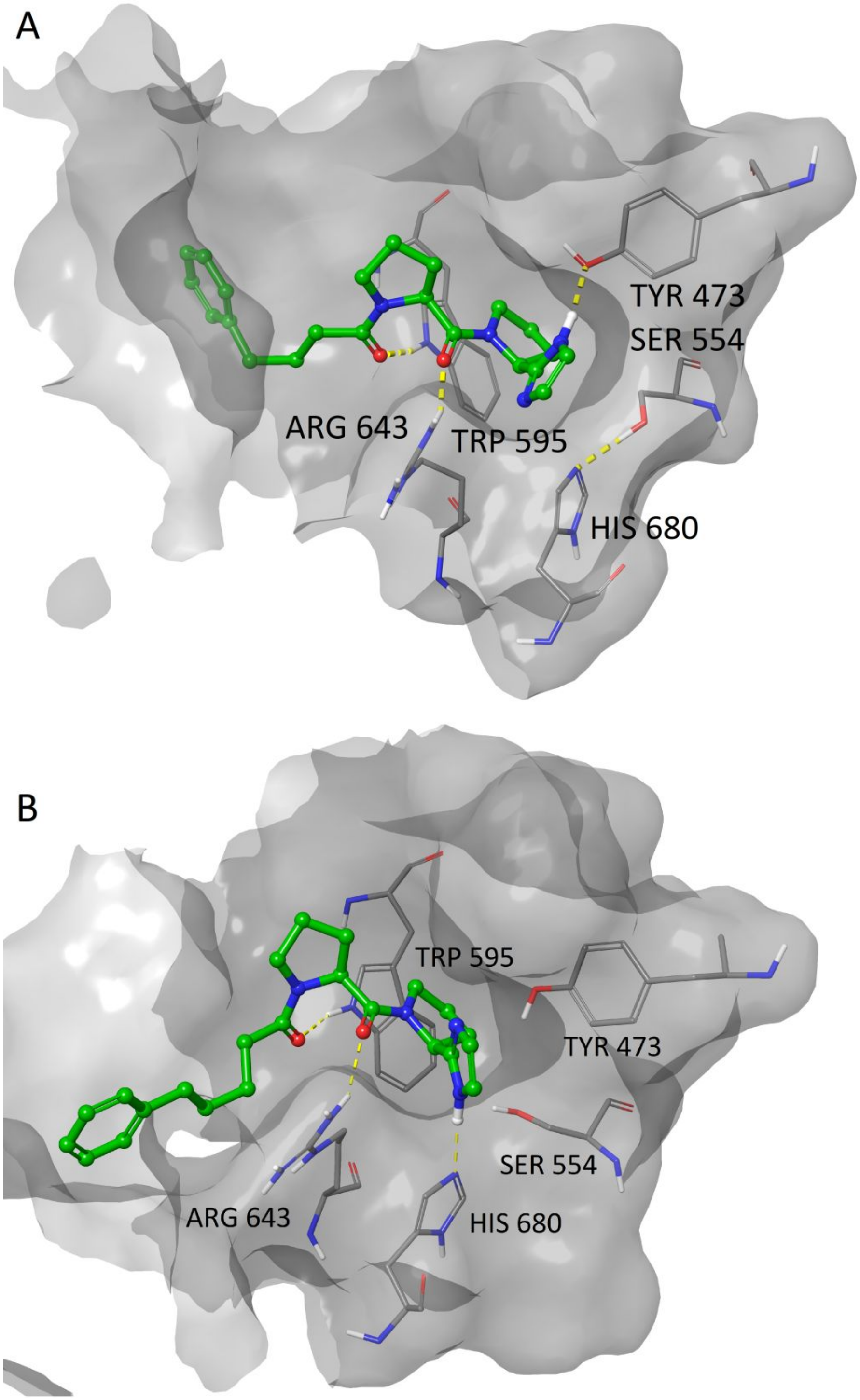

Figure S3. Induced fit docking (A) alternative pose for **27a** and (B) pose for **28a** at the proteolytic active site of PREP (PDB: 3DDU). Hydrogen bonds are shown as yellow dashed lines.

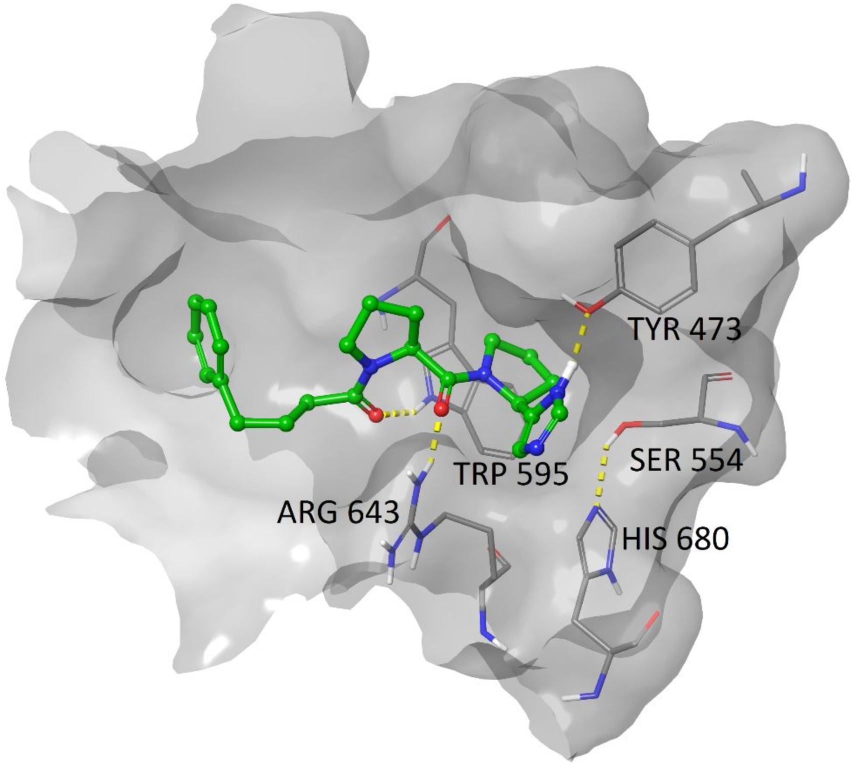

Figure S4. Induced fit docking pose for **29a** at the proteolytic active site of PREP (PDB: 3DDU). Hydrogen bonds are shown as yellow dashed lines.

UPLC-MS figures

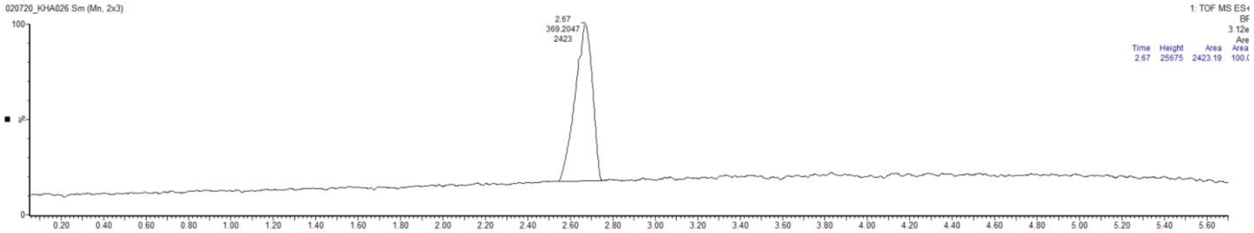

Figure S5. UPLC-MS trace from **15a**.

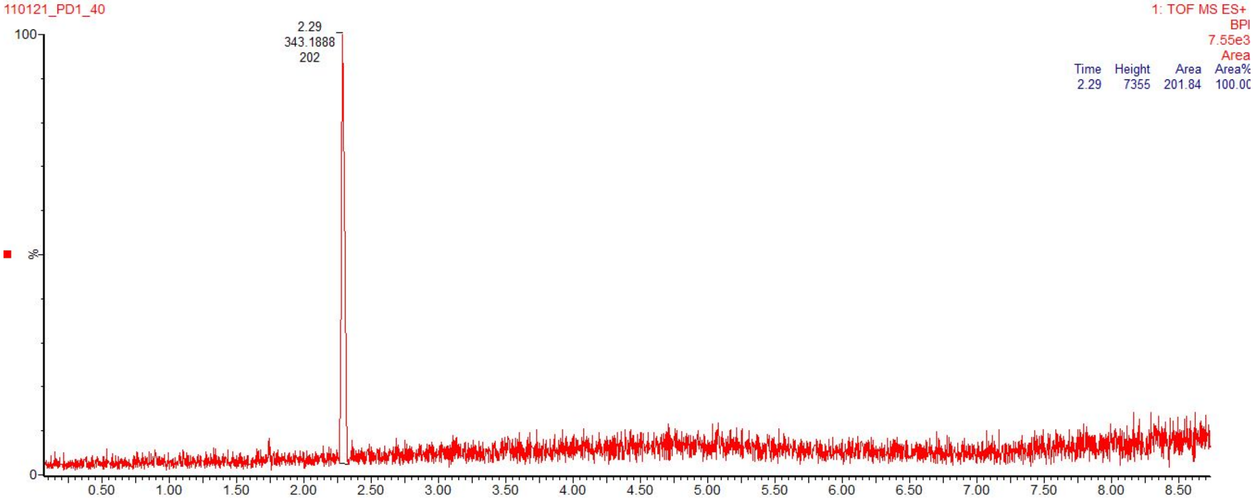

Figure S6. UPLC-MS trace from **15b**.

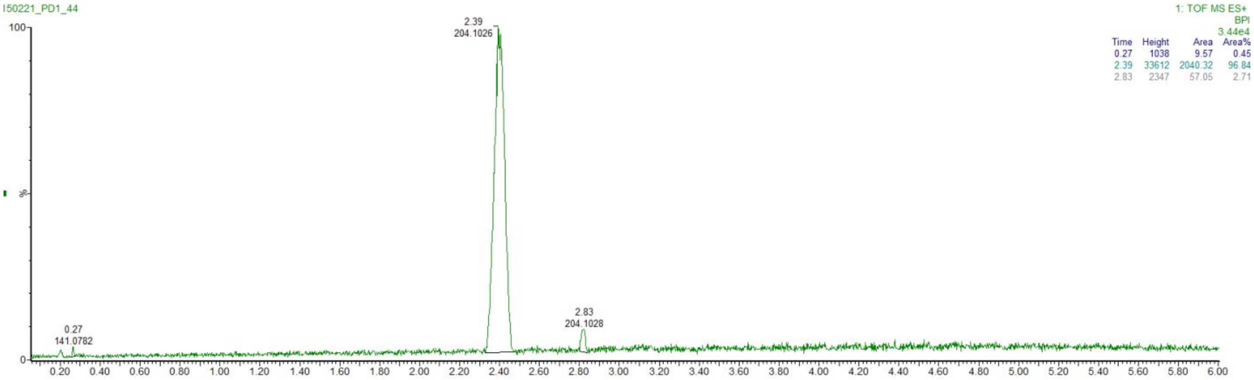

Figure S7. UPLC-MS trace from **15c**.

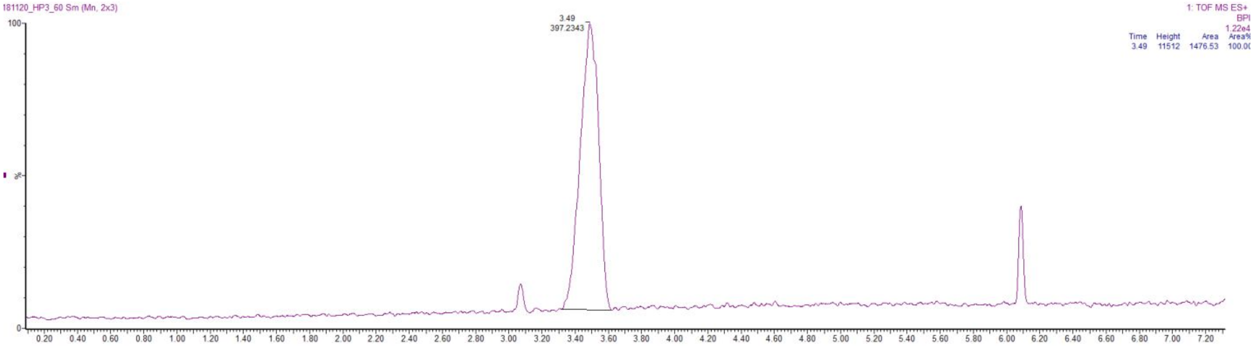

Figure S8. UPLC-MS trace from **16a**.

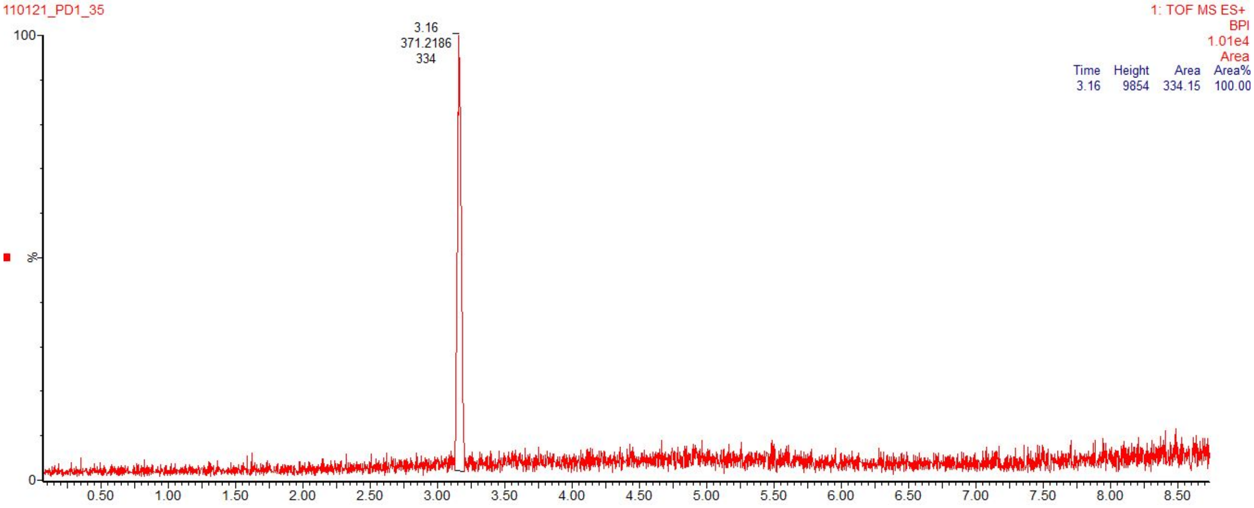

Figure S9. UPLC-MS trace from **16b**.

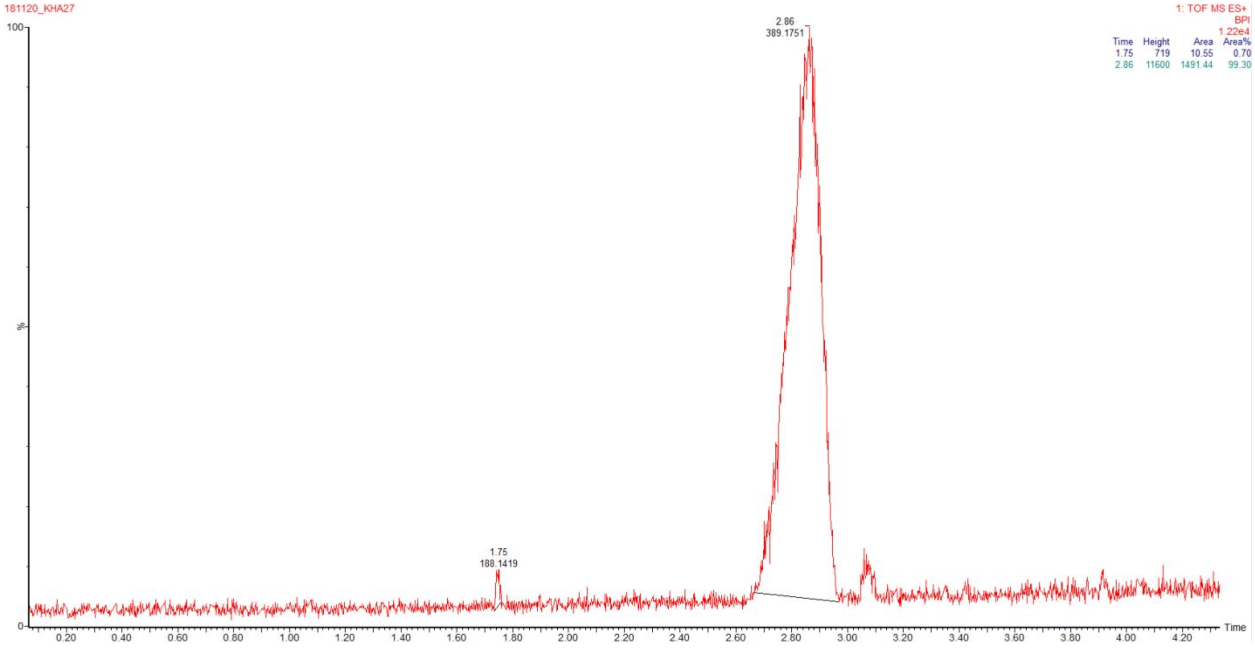

Figure S10. UPLC-MS trace from **17a**.

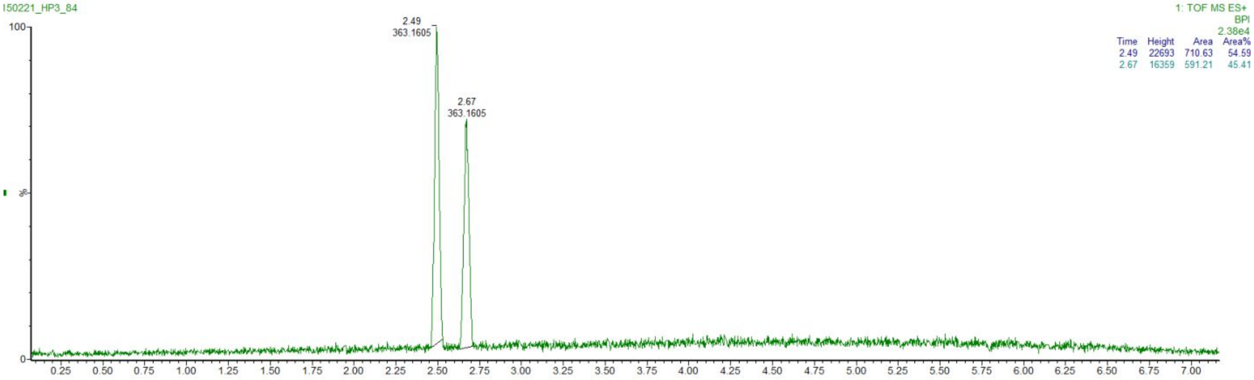

Figure S11. UPLC-MS trace from **17b**.

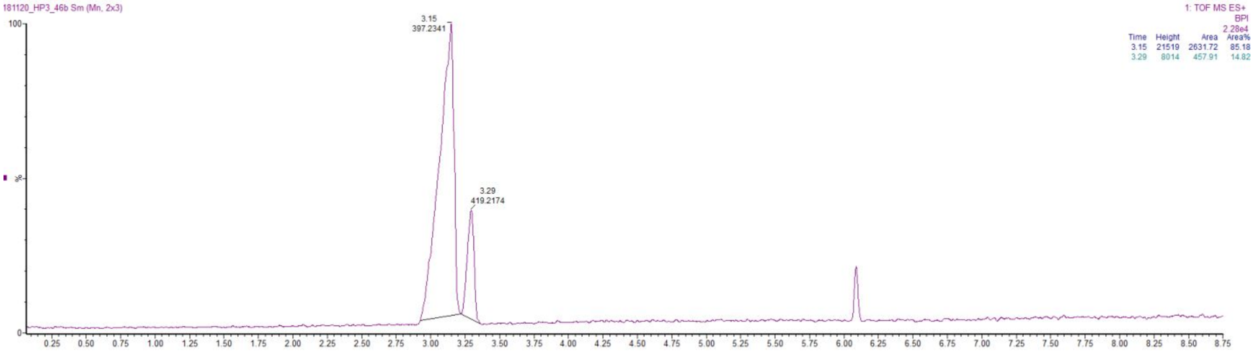

Figure S12. UPLC-MS trace from **18a**. Minor peak from other regioisomer. Mass 419 corresponds to the Na salt of **18a**.

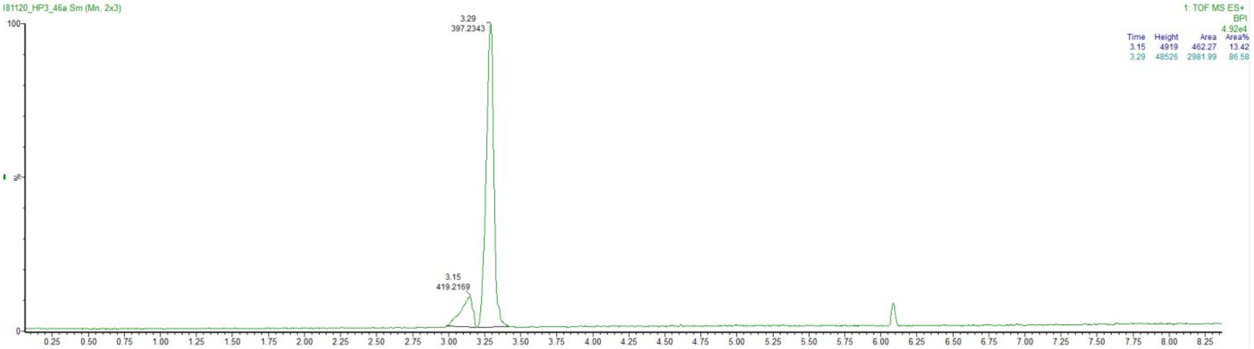

Figure S13. UPLC-MS trace from **19a**. Minor peak from other regioisomer. Mass 419 corresponds to the Na salt of **19a**.

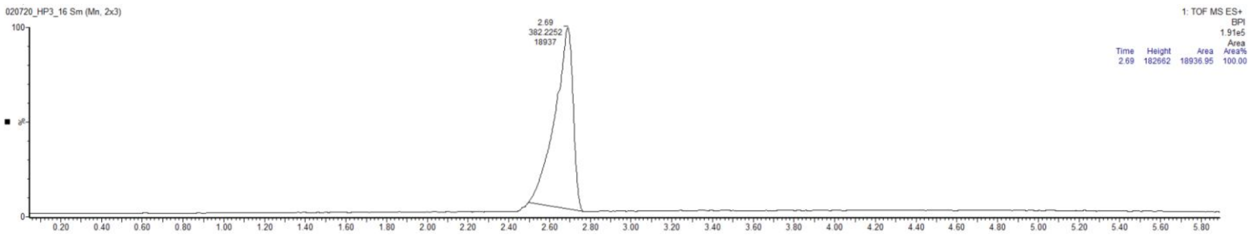

Figure S14. UPLC-MS trace from **20a**.

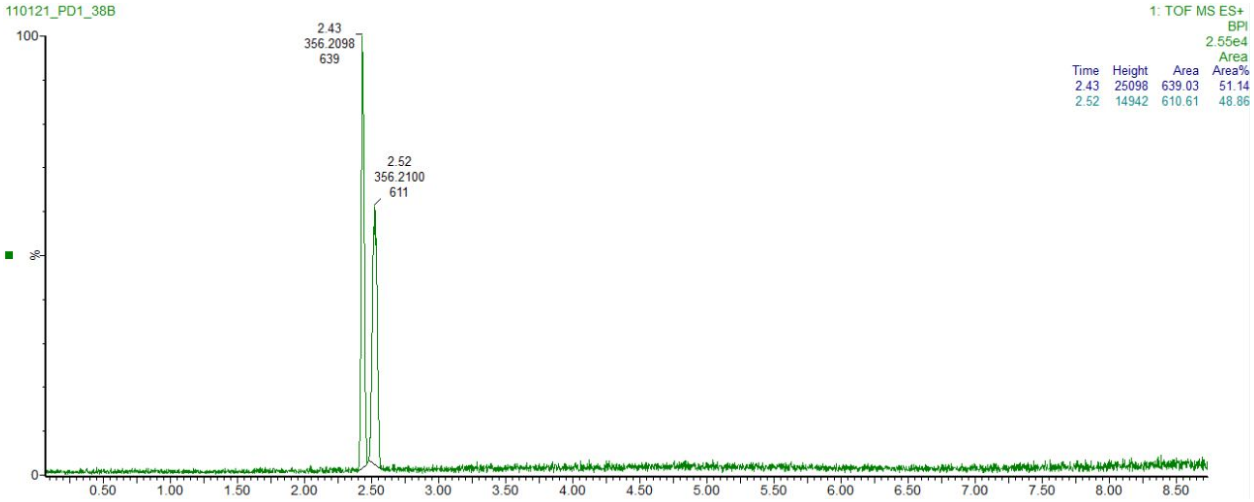

Figure S15. UPLC-MS trace from **20b**.

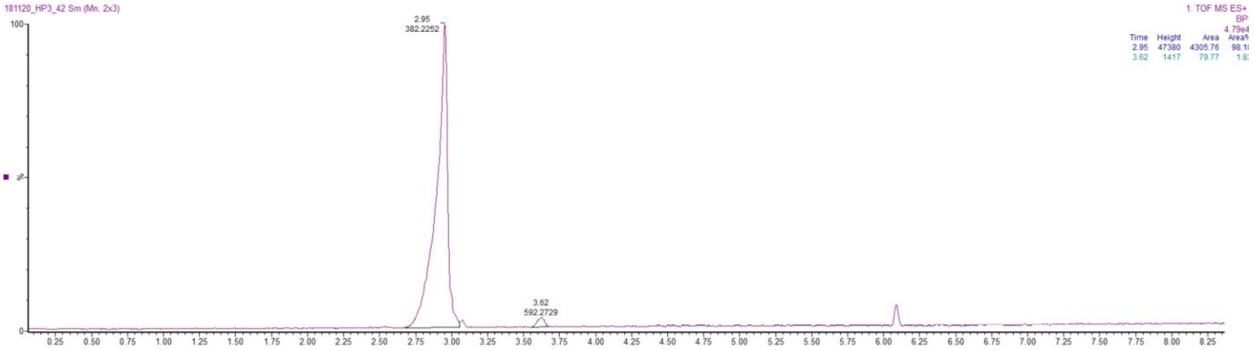

Figure S16. UPLC-MS trace from **25a**.

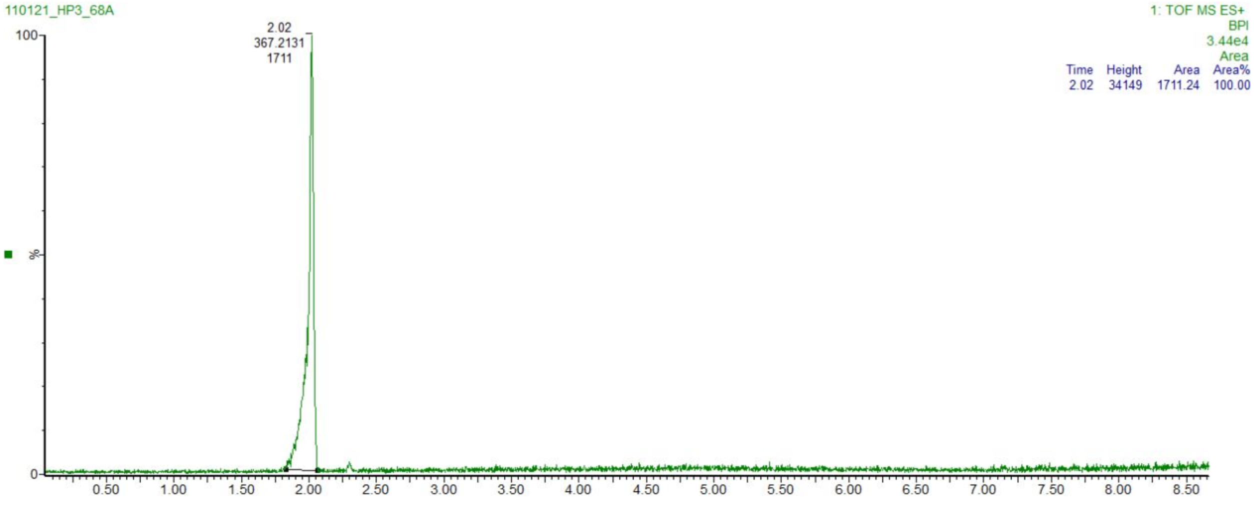

Figure S17. UPLC-MS trace from **26a**.

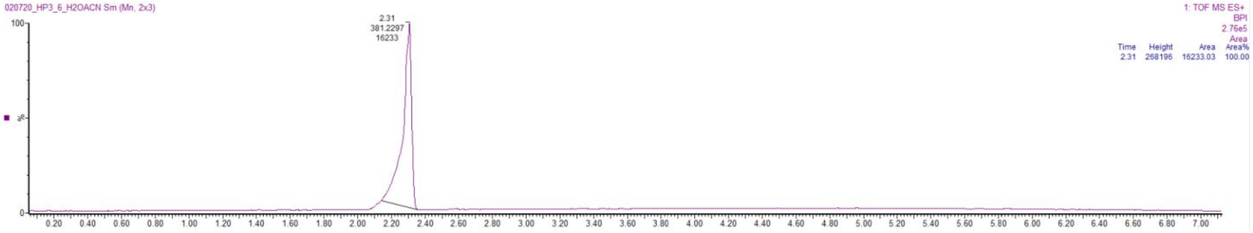

Figure S18. UPLC-MS trace from **27a**.

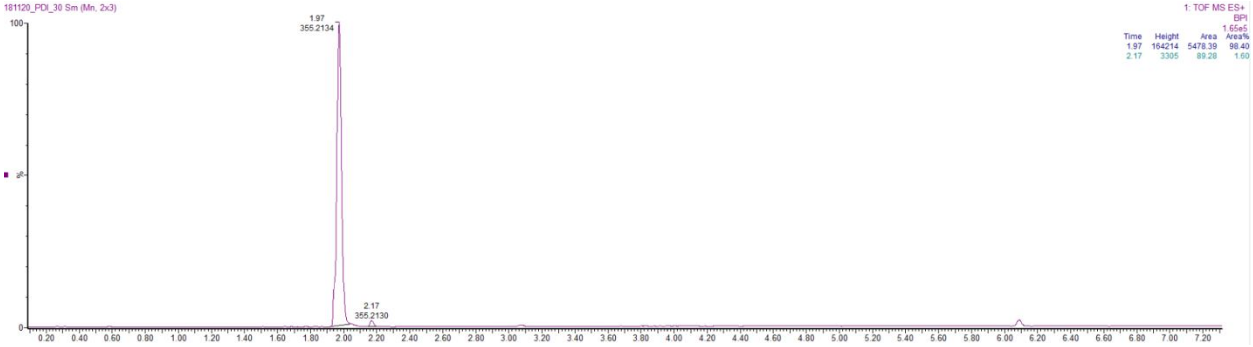

Figure S19. UPLC-MS trace from **27b**.

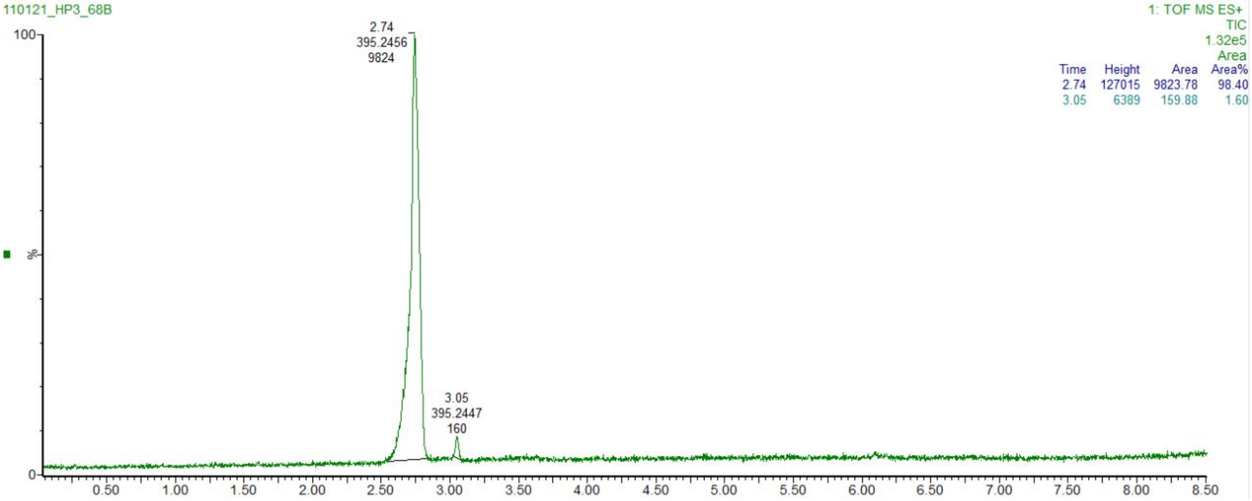

Figure S20. UPLC-MS trace from **28a**.

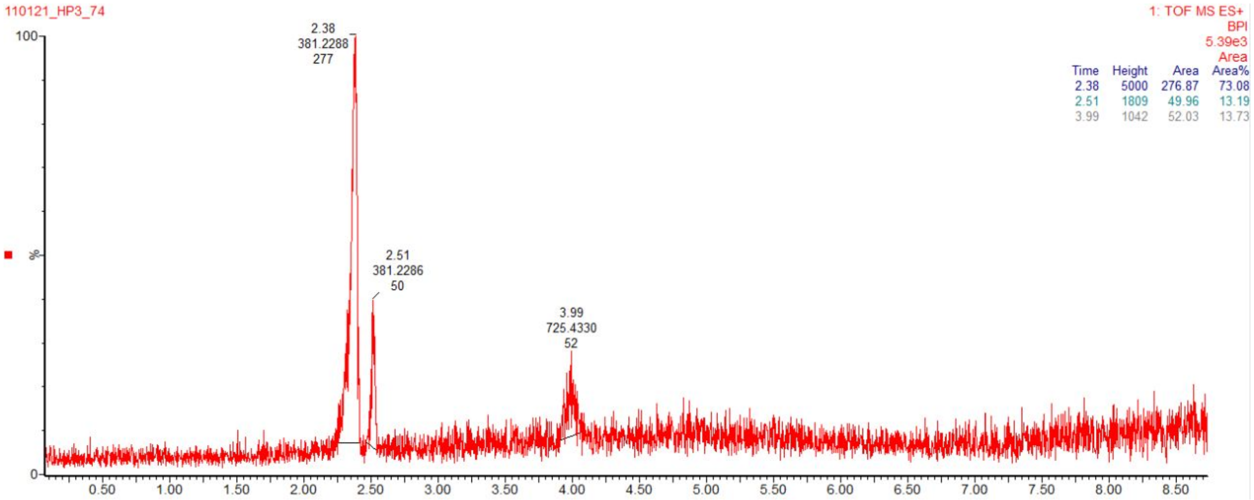

Figure S21. UPLC-MS trace from **29a**.

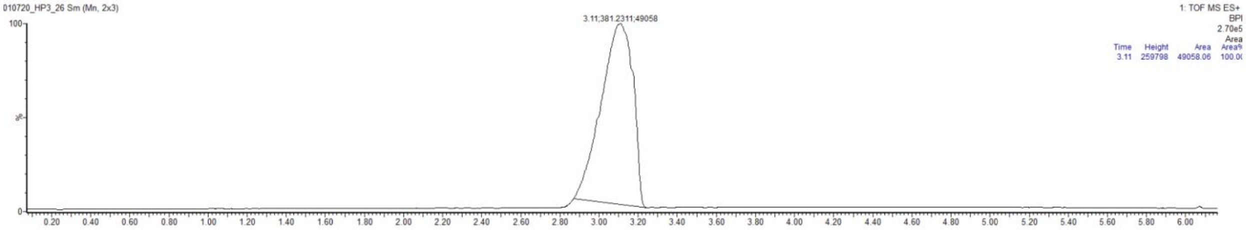

Figure S22. UPLC-MS trace from **30a**.

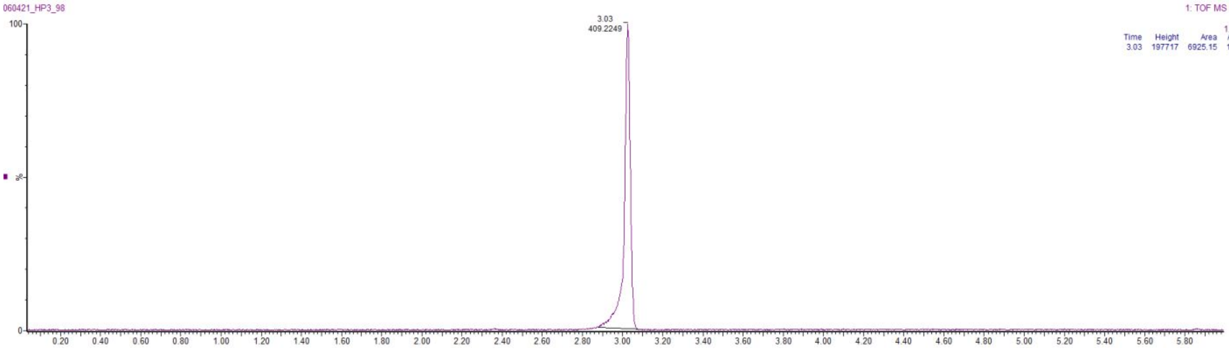

Figure S23. UPLC-MC trace from **2a**.

References

1. Kilpelainen, T. P.; Tyni, J. K.; Lahtela-Kakkonen, M. K.; Etelainen, T. S.; Myohanen, T. T.; Wallen, E. A. A. Tetrazole as a Replacement of the Electrophilic Group in Characteristic Prolyl Oligopeptidase Inhibitors. *ACS Med Chem Lett* **2019,** *10* (12), 1635-1640.
2. Tsutsumi, S.; Okonogi, T.; Shibahara, S.; Ohuchi, S.; Hatsushiba, E.; Patchett, A. A.; Christensen, B. G. Synthesis and structure-activity relationships of peptidyl alpha-keto heterocycles as novel inhibitors of prolyl endopeptidase. *J Med Chem* **1994,** *37* (21), 3492-3502.
3. Venäläinen, J. I.; Juvonen, R. O.; Forsberg, M. M.; Garcia-Horsman, A.; Poso, A.; Wallen, E. A.; Gynther, J.; Männistö, P. T. Substrate-dependent, non-hyperbolic kinetics of pig brain prolyl oligopeptidase and its tight binding inhibition by JTP-4819. Biochem Pharmacol **2002**, 64 (3), 463-471.
4. Svarcbahs, R.; Jäntti, M.; Kilpeläinen, T.; Julku, U. H.; Urvas, L.; Kivioja, S.; Norrbacka, S.; Myöhänen, T. T. Prolyl oligopeptidase inhibition activates autophagy via protein phosphatase 2A. *Pharmacol Res* **2020,** *151*, 104558.
5. Kaizuka, T.; Morishita, H.; Hama, Y.; Tsukamoto, S.; Matsui, T.; Toyota, Y.; Kodama, A.; Ishihara, T.; Mizushima, T.; Mizushima, N. An Autophagic Flux Probe that Releases an Internal Control. *Mol Cell* **2016,** *64* (4), 835-849.
6. Schrödinger Release 2020-4, Maestro
7. Haffner, C. D.; Diaz, C. J.; Miller, A. B.; Reid, R. A.; Madauss, K. P.; Hassell, A.; Hanlon, M. H.; Porter, D. J.; Becherer, J. D.; Carter, L. H. Pyrrolidinyl pyridone and pyrazinone analogues as potent inhibitors of prolyl oligopeptidase (POP). *Bioorg Med Chem Lett* **2008,** *18* (15), 4360-4363.
8. Schrödinger Release 2020-4, Protein Preparation Wizard
9. Schrödinger Release 2020-4, Prime
10. Schrödinger Release 2020-4, Epik
11. Olsson, M. H. M.; Søndergaard, C. R.; Rostkowski, M.; Jensen, J. H. PROPKA3: Consistent Treatment of Internal and Surface Residues in Empirical pKa Predictions. *J Chem Theory Comput* **2011,** *7* (2), 525-537.
12. Schrödinger Release 2020-4, LigPrep
13. Schrödinger Release 2020-4, Glide
14. Schrödinger Release 2020-4, Induced Fit Docking protocol
15. Kaszuba, K.; Róg, T.; Danne, R.; Canning, P.; Fülöp, V.; Juhász, T.; Szeltner, Z.; St. Pierre, J. F.; García-Horsman, A.; Männistö, P. T.; Karttunen, M.; Hokkanen, J.; Bunker, A., Molecular dynamics, crystallography and mutagenesis studies on the substrate gating mechanism of prolyl oligopeptidase. *Biochimie* **2012,** *94* (6), 1398-1411.
